# Reduced Basal Nitric Oxide Production Induces Precancerous Mammary Lesions via ERBB2 and TGFβ

**DOI:** 10.1038/s41598-019-43239-x

**Published:** 2019-04-30

**Authors:** Gang Ren, Xunzhen Zheng, Matthew Bommarito, Samantha Metzger, Yashna Walia, Joshua Letson, Allen Schroering, Andrea Kalinoski, David Weaver, Christopher Figy, Kam Yeung, Saori Furuta

**Affiliations:** 1 University of Toledo Health Science Campus, College of Medicine and Life Sciences, Department of Cancer Biology, 3000 Arlington Ave, MS1010 Toledo, OH USA; 2 University of Toledo Health Science Campus, College of Medicine and Life Sciences, Department of Surgery, 3000 Arlington Ave, MS1008 Toledo, OH USA

**Keywords:** Breast cancer, Mechanisms of disease, Breast cancer

## Abstract

One third of newly diagnosed breast cancers in the US are early-stage lesions. The etiological understanding and treatment of these lesions have become major clinical challenges. Because breast cancer risk factors are often linked to aberrant nitric oxide (NO) production, we hypothesized that abnormal NO levels might contribute to the formation of early-stage breast lesions. We recently reported that the basal level of NO in the normal breast epithelia plays crucial roles in tissue homeostasis, whereas its reduction contributes to the malignant phenotype of cancer cells. Here, we show that the basal level of NO in breast cells plummets during cancer progression due to reduction of the NO synthase cofactor, BH_4_, under oxidative stress. Importantly, pharmacological deprivation of NO in prepubertal to pubertal animals stiffens the extracellular matrix and induces precancerous lesions in the mammary tissues. These lesions overexpress a fibrogenic cytokine, TGFβ, and an oncogene, ERBB2, accompanied by the occurrence of senescence and stem cell-like phenotype. Consistently, normalization of NO levels in precancerous and cancerous breast cells downmodulates TGFβ and ERBB2 and ameliorates their proliferative phenotype. This study sheds new light on the etiological basis of precancerous breast lesions and their potential prevention by manipulating the basal NO level.

## Introduction

One in eight women in the United States is diagnosed with breast cancer in their lifetime, making it the second leading cause of cancer death among women^1^. Of the 300,000 new breast cancer cases diagnosed in the US each year, nearly 30% are early-stage lesions including hyperplasia, atypia and *in situ* cancers^1,2^. Despite being precancerous, early-stage breast lesions are the precursor of invasive cancers, and over 40% of them could progress to invasive cancer if left untreated^2^. Because of the dramatic increase in the incidence as well as etiological and therapeutic uncertainties, early-stage breast lesions have become a major clinical challenge over the past decades^2^.

To understand the basis of early-stage breast lesions, a number of recent studies report the causative roles of different breast cancer risk factors, especially, those that are independent of genetic predisposition and can therefore be modified. These risk factors include a high-fat diet, moderate to heavy alcohol intake, smoking, low physical activity, diabetes, obesity and hypertension^3–9^. Interestingly, these different risk factors are commonly linked to aberrant production of nitric oxide (NO)^10–16^, a bioactive signaling molecule produced throughout the body. This led us to hypothesize that abnormal levels of NO in the breast might contribute to formation of precancerous breast lesions.

NO is produced by NO synthases 1–3 (NOS 1–3) using arginine as the substrate to exert pleiotropic functions. Its bioactivities can dramatically differ depending on the concentration, timing and context^17–20^. In canonical signaling, physiological stress promotes the production of large amounts of NO, particularly by the inducible NOS2, triggering proper functions of specialized cells including neurons, muscles, endothelia and immune cells^21^. Conversely, under the unstressed, normal physiological conditions, NO is produced at the basal steady-state level by the constitutive NOS1 (neuronal) and NOS3 (endothelial) in diverse cell types contributing to tissue morphogenesis, homeostasis and tissue-specific functions^22–25^. In mammary glands, NOS-1 and -3 are constitutively expressed and are elevated during pregnancy^26–28^, while NO production increases in the postpartum period^29,30^. This not only promotes alveolar (milk-producing unit) development, blood flow and nutrient uptake for milk production^31,32^, but also facilitates milk ejection^27,33^. Moreover, NO is secreted into the breast milk as an essential component for immunity and neonatal growth^34^.

In diseased states including cancer, however, NO production is often dysregulated. Some studies report that NO production increases during cancer progression^18,35,36^, while others report the opposite^24,37,38^. Thus, too much or too little NO might equally contribute to disease pathogenesis^39,40^. NO’s activities in cancer are also complex and contradictory^41^. NO can exert dichotomous effects on diverse cellular processes including proliferation, apoptosis, migration, invasion and angiogenesis. Such variations depend on NO’s concentration, context, timing, microenvironment, cancer type and stage^18,20,41–43^. For example, NO activates pro-tumoral signals (ERK and HIF1-α) at lower concentrations (<300 nM), but activates anti-tumoral signals (p53) at higher concentrations (>300 nM)^42^. Furthermore, NO could be produced by cancer cells or cancer-associated macrophages (M1 type), leading to either pro- or anti-tumoral effects^41,44^. This intricacy has led to conflicting reports and a notion that NO plays a double-edged role as both a cancer-promoter and -inhibitor^17,18,20,45^.

To make matters more complicated, in many diseases including cancer, NOS might be dysfunctional due to deprivation of the redox-sensitive cofactor, tetrahydrobiopterin (BH_4_), while being under oxidative stress. In this state, NOS fails to form the functional homodimer to produce NO and remains as monomers^46^. NOS monomers then produce superoxide instead of NO (“*uncoupled*”), exacerbating the disease pathogenesis^47–50^. Such alterations in the functionality of NOS might, at least in part, explain why NOS, in particular NOS2 isoform, is preferentially upregulated in many types of cancers^51,52^.

To support our hypothesis that aberrant NO production is involved in normal-to-precancerous progression of the breast epithelia, we have recently reported that non-malignant mammary epithelial cells (MECs) produce NO at the basal steady-state level upon interacting with the laminin-rich extracellular matrix (ECM)^24,53^. Such NO production is critical for the establishment of mammary tissue architecture and homeostasis^24^. On the other hand, in the malignant counterpart derived from the same patient, NO production is defective, contributing to formation of proliferative, disorganized structures in 3D ECM cultures^24,53^.

In the present study, we investigate whether aberrant NO production contributes to the formation of precancerous breast lesions from normal mammary epithelia using cell lines of a breast cancer progression series as well as animal models. Here, we show that the basal level of NO production in cultured MECs plummets along with cancer progression. This was primarily due to reduction of the NOS cofactor, BH_4_, under increased oxidative stress (NOS uncoupling), but was independent of NOS levels. Importantly, pharmacological deprivation of NO in developing mouse mammary glands led to the formation of multiple (peripheral) papillomas (precancerous mammary lesions)^54–58^ and desmoplastic ECM in all the animals tested. NO-deprived mammary glands exhibited overactivation of an oncogene, ERBB2, and a fibrogenic cytokine, TGFβ. These glands also highly expressed senescence markers, reported to be prevalent in precancerous lesions^59–61^. In addition, NO-deprived mammary glands displayed bi-lineage phenotype and stem cell-like properties, which are also reported to be prevalent in precancerous lesions^62–64^. Consistently, application of the BH_4_ precursor (sepiapterin) or a NO donor (SNAP or GSNO) to precancerous and cancerous breast cells normalized NO levels, suppressed TGFβ and ERBB2 signals and ameliorated proliferative phenotype. Our results unravel the critical contribution of deficient basal NO production to the pathogenesis of precancerous breast lesions.

## Results

### Basal level of NO production in cultured mammary epithelial cells plummets during normal-to-precancerous progression

We previously demonstrated that non-malignant MECs produce the basal steady-state level of NO immediately after contacting laminin, but not collagen, in the extracellular matrix (ECM)^24^. This observation is in agreement with a notion that NO production is largely influenced by chemical and mechanical properties of the tissue microenvironment such as the ECM^53,65–67^.

In our previuos studies, to test whether basal NO production is altered in breast cancer progression, we used the HMT-3522 breast cancer progression series composed of non-malignant S1 and malignant T4-2 cells^68,69^. S1 was derived from a benign mammary fibrocystic lesion and became spontaneously immortalized in culture^68,70–72^. S1 cells retain non-malignant characteristics, requiring EGF to grow in culture and being unable to form tumors in nude mice^68,71,72^. (The use of S1 cells is restricted to passages below 60 because of genotypic drift at higher passages^71,72^.) S1 cells were utilized to generate the malignant counterpart (HMT-3522-T4-2) by withdrawing EGF from the growth medium and serially transplanting cells into animals, without overexpressing oncogenes^72,73^. We showed that basal NO production in response to laminin is pronounced in non-malignant S1, but is impaired in malignant T4-2 cells^24,53^. This observation was confirmed using normal vs. malignant human breast tissues^24^.

In the present study, we attempted to test whether basal NO production is altered stepwise during malignant progression of MECs. We utilized the MCF10A breast cancer progression series, composed of four isogenic cell lines: MCF10A, AT1, DCIS.COM and CA1d^74–80^. MCF10A, harboring many characteristics of normal breast epithelium, was derived from a mammary fibrocystic lesion and was spontaneously immortalized in culture^74^. AT1, showing the features of atypical hyperplasia, was generated by transfecting mutant H-Ras into MCF10A cells and serially transplanting them into nude mice^75,76^. AT1 cells do not initially grow out as carcinomas in nude mice; however, within 2 years 25% of them become cancerous^76^. DCIS.COM, showing dysplastic comedo DCIS phenotype, was derived from cells of a hyperplastic lesion formed by AT1 cells in nude mice^77^. DCIS.COM cells do not initially grow out as carcinomas in nude mice; however, after 9 weeks half of them form tumors^77^. CA1d was derived from a tumor formed by AT1 cells after one year in animals^78^. 100% of CA1d cells form metastatic tumors in nude mice^78^.

**Figure 1:**
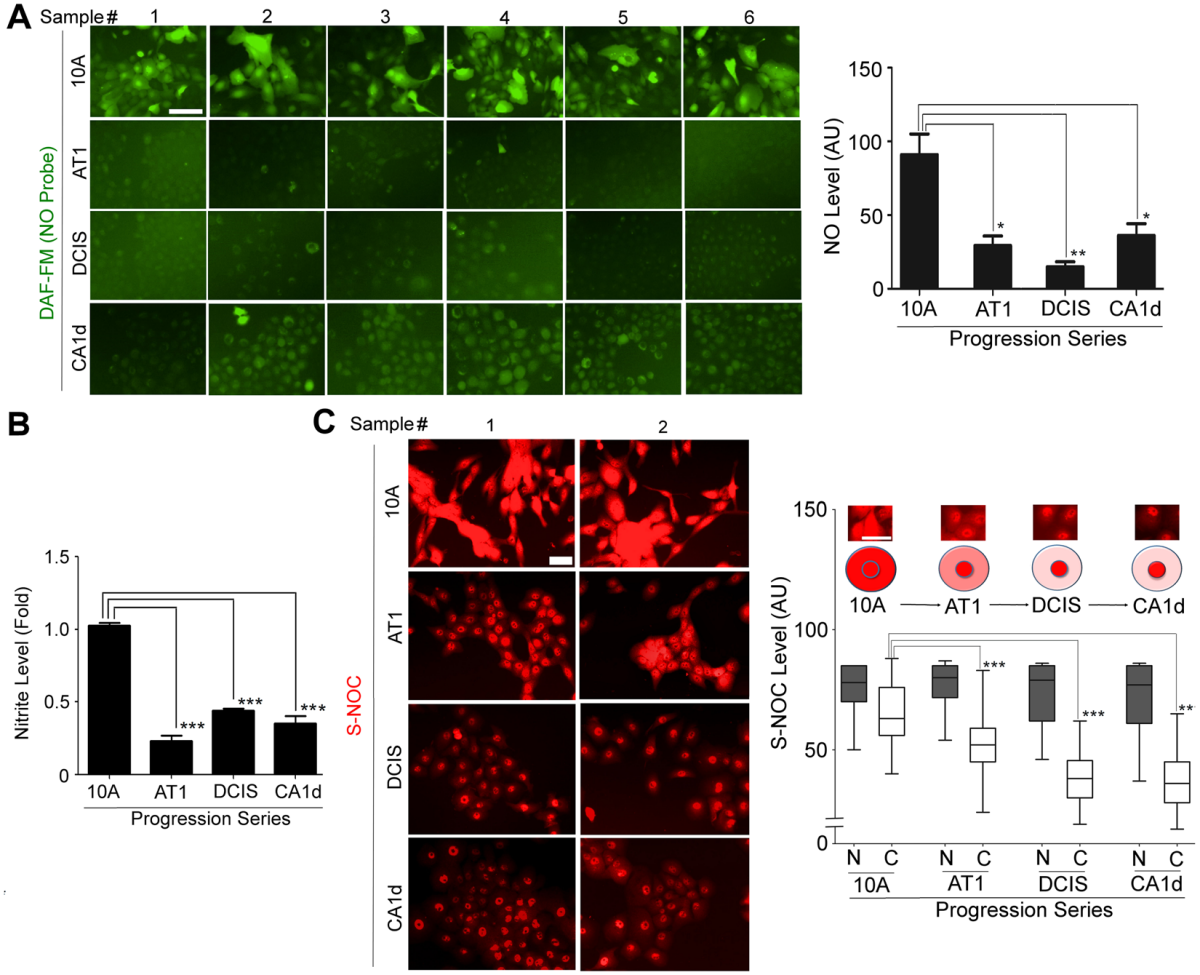
Non-malignant mammary epithelial cells produce the basal level of nitric oxide (NO) in 3D ECM cultures, whereas the level plummets along with breast cancer progression. (**A**) (Left) Cell lines of MCF10A breast cancer progression series (10A; non-malignant; AT1: premalignant; DCIS: ductal carcinoma *in situ*; and CA1d: malignant)^74–80^ cultured with 5% Matrigel drip and stained with the fluorescent NO probe DAF-FM DA. (Right) Quantification of DAF-FM DA signal/cell in the series. (See DAF-FM DA signals of a panel of normal vs. cancerous MECs in Supplementary Fig. 1A,B). (**B**) The level of NO metabolite nitrite in the conditioned media of the progression series. (**C**) (Left) Cell lines of the progression series stained with an antibody against S-nitrosocysteine (S-NOC). (Right) Quantification of S-NOC level in the nucleus (N) vs. cytosol (C) of the series. AU: arbitrary unit. Error bars: mean ± STDEV. *p < 0.05; **p < 0.01; and ***p < 0.001.

We pulse-treated these cell lines with a drip of reconstituted laminin-rich ECM (lrECM, a.k.a. Matrigel) and determined the level of intracellular NO using a fluorescent probe DAF-FM^24,81^. While non-malignant (MCF10A) cells produced an appreciable level of DAF FM signal, the level was dramatically (~−60%) reduced in precancerous (AT1) cell and remained low in cells with more advanced stages (DCIS and CA1d) (Fig. 1A). As a complementary approach, we measured the level of the NO metabolite (nitrite) in the conditioned media of cells cultured in 3D lrECM using a fluorescent probe DAN^24,82^ and observed similar results (Fig. 1B). Furthermore, we analyzed the level of S-nitrosocysteine (SNOC, a NO-dependent protein modification) as an indicator of NO production. Consistently, the cytosolic, but not nuclear, SNOC level progressively declined along with cancer progression (Fig. 1C).

To test the generality of this phenomenon, we compared basal NO production in response to lrECM among a panel of normal/non-malignant vs. cancerous breast cell lines using the NO probe DAF-FM. The specificity of the signal was confirmed by quenching it with the NOS inhibitor, L-NAME (2.5 mM), an L-arginine analog that competitively inhibits the substrate binding to NOS (IC_50_ ~ 70 μM)^83^ [and to arginase (IC_50_ ~ 27 mM)^84,85^]. DAF-FM signals were generally much higher in normal/non-malignant MECs than breast cancer cells (Supplementary Fig. 1A,B), consistent with previous reports^24,53,86^. These results altogether demonstrate that the levels of basal NO become lower in MECs along with cancer progression.

### NOS-1 and -3 levels remain high, while NOS-2 level remains low, in all cell lines of the breast cancer progression series

**Figure 2:**
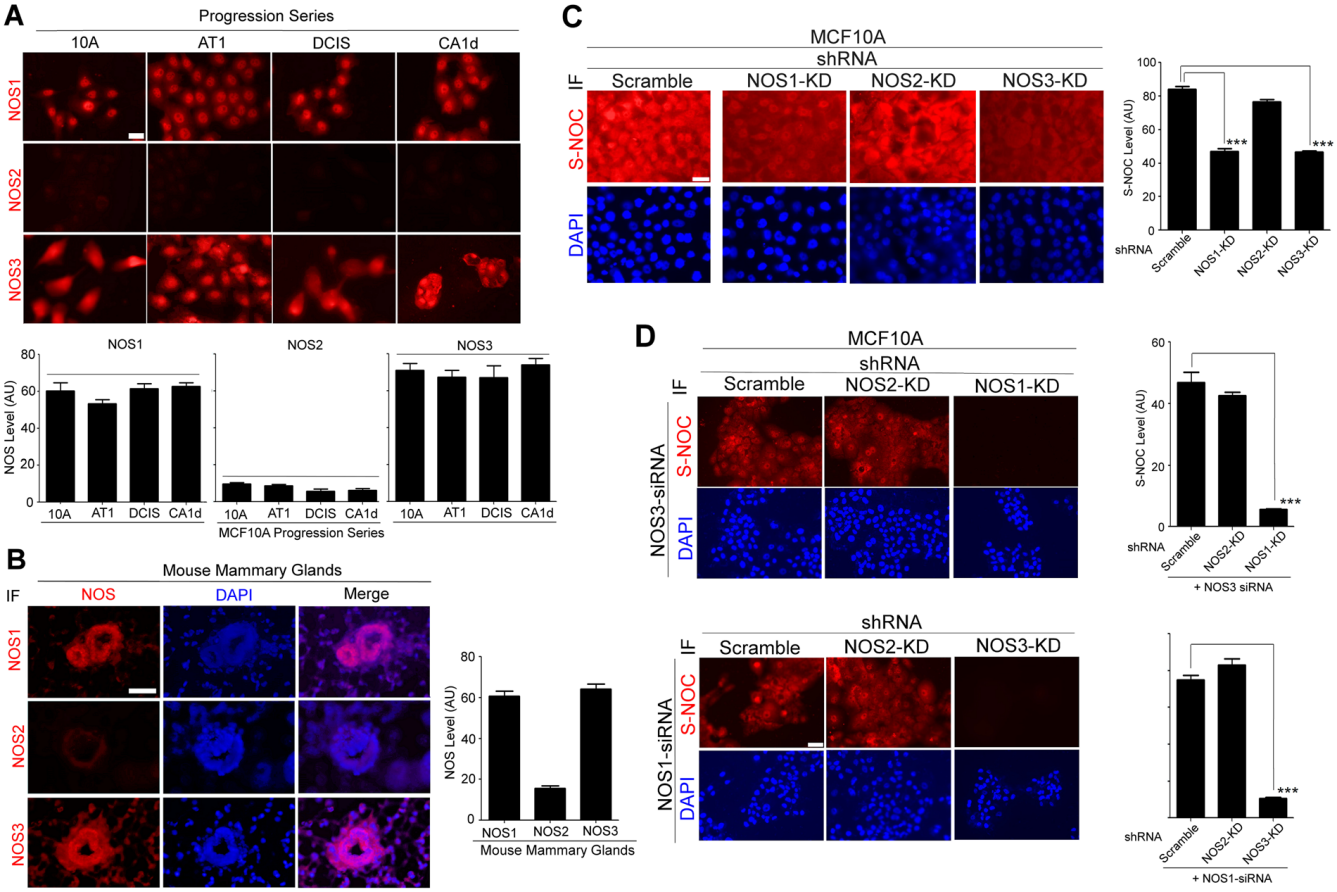
Nitric oxide synthase (NOS)-1 and -3 are involvd in production of basal NO in MECs. (**A**) (Top) NOS-1, -2 or -3 staining of the MCF10A breast cancer progression series. Scale bars: 20 μm. (Bottom) Quantification of NOS signal/cell. AU: Arbitrary unit. (**B**) (Left) NOS-1, -2 or -3 staining of mouse mammary glands (10 weeks old, virgin female). Nuclei were counterstained with DAPI (blue). Scale bars: 50 μm. (Right) Quantification of NOS signal/cell. (**C**) (Left) S-NOC staining of MCF10A cells expressing scramble, NOS-1, -2 or -3 shRNA. Nuclei were counterstained with DAPI (blue). Scale bars: 20 μm. (Right) Quantification of SNOC signal/cell. (**D**) S-NOC staining of MCF10A cells with double knockdown (KD) of NOS-1 and -3 using different combinations of shRNA and siRNA. (Top 2 rows, left) S-NOC staining of MCF10A cells expressing scramble, NOS-2 or -1 shRNA, which were further transfected with NOS-3 siRNA. (Top right) Quantification of S-NOC signal/cell. (Bottom 2 rows, left) S-NOC staining of MCF10A cells expressing scramble, NOS-2 or -3 shRNA, which were further transfected with a NOS-1 siRNA. (Bottom right) Quantification of S-NOC signal/cell. Note that while KD of NOS-1 or -3 alone partially inhibited SNO, double KD of NOS-1/NOS-3 abrogated SNOC. Scale bars: 50 μm.

NO is produced by three isoforms of NO synthases (NOS 1–3)^21^. To determine which isoform is involved in NO production in MECs, we examined the level of each NOS isoform in the MCF10A progression series. In non-malignant MCF10A cells, NOS-1 and -3 were both expressed at high levels, whereas NOS-2 was undetectable (Fig. 2A). We then examined the level of each NOS isoform in normal mouse mammary glands. The expression patterns of NOS isoforms were the same as those in MCF10A, consistent with previous reports (Fig. 2B)^26–28^. Interestingly, throughout the entire progression series, NOS expression patterns did not change. NOS-1 and -3 remained high, while NOS-2 remained low (Fig. 2A). This result suggests that NOS levels have no relevance to reduction of basal NO level in cell lines of breast cancer progression series.

### Basal level of NO production in mammary epithelial cells is mediated by NOS-1 and -3

To confirm that NOS-1 and -3 are indeed involved in basal NO production in MECs, we nearly depleted each isoform with a specific shRNA in non-malignant MCF10A cells (Supplementary Fig. 2A), which were shown to produce the highest level of NO among all cell lines of the progression series (Fig. 1A). We then examined which NOS depletion had impaired NO production by comparing SNOC levels. Single knock-down (KD) of either neuronal NOS1 or endothelial NOS3 partially reduced SNOC level, whereas KD of inducible NOS2 had no effect (Fig. 2C). Conversely, simultaneous inhibition of NOS3 in NOS1-KD cells or NOS1 in NOS3-KD cells with a specific siRNA (Fig. 2D) or with a small molecule inhibitor (Supplementary Fig. 2B) almost abrogated SNOC signal. These results suggest that NOS-1 and -3 (but not NOS-2) equally participate in production of the basal level of NO in MECs.

### Reduced NO production in cell lines of breast cancer progression series correlates with increased acidity and oxidative stress that depletes the NOS cofactor BH_4_

We further examined why NO production dramatically declines in cell lines of the breast cancer progression series, while NOS levels remain unchanged (Figs 1A, 2A). We postulated that this might be attributed to certain physiological traits associated with cancer progression that could debilitate NOS functions. To test this, we measured the intracellular pH and oxidative stress (superoxide and total reactive oxygen species). Acidification and oxidative stress are hallmarks of cancer metabolism^87^ and also known to attenuate NOS functions^49,88–90^. As expected, the acidity and oxidative stress were significantly elevated along with cancer progression (Fig. 2A–C).

In particular, oxidative stress has been shown to deplete the essential NOS cofactor, BH_4_. BH_4_ helps tether two NOS monomers to form the functional NOS homodimer, allowing for coupling two reactions required for NO production: (1) reduction of molecular oxygen and (2) oxidation of arginine^91^. When BH_4_ (or arginine) is deficient_,_ however, NOS remains as monomers, and these two reactions are uncoupled, releasing superoxide (from the former reaction only) instead of producing NO —a phenomenon termed ‘*NOS uncoupling’*^49,88^.

**Figure 3:**
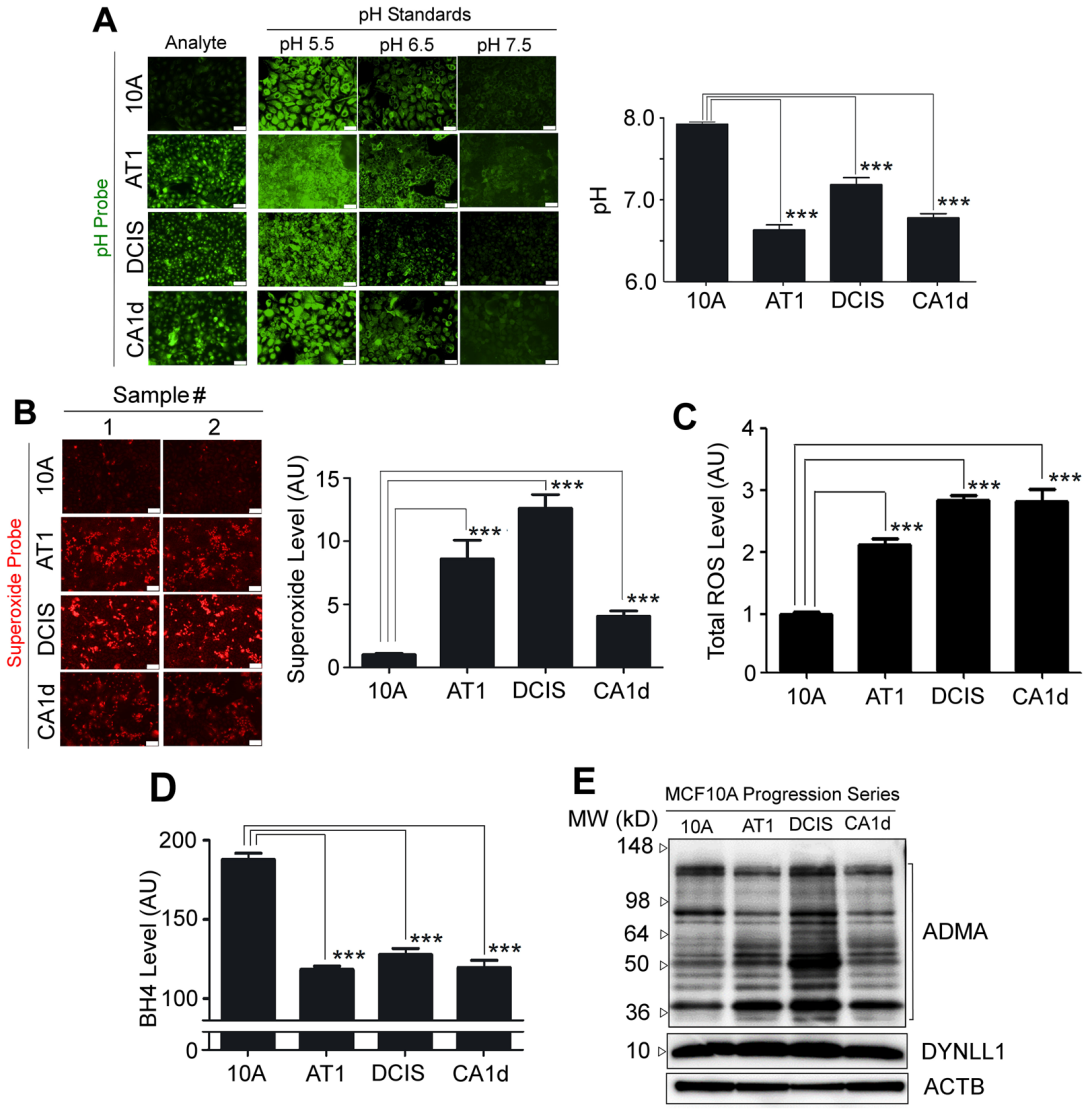
Decrease of the basal NO level along with breast cancer progression parallels the dramatic increase in acidity and oxidative stress and the decline of the NOS cofactor BH_4_. (**A**) Cells of the MCF10A progression series stained with pH probe. (Left) Cells treated with pH standard (pH 5.5–7.5) are shown on the right, whereas the analytes are shown on the left. Scale bars: 100 μm. (Right) pH level of the series. Error bars: mean ± STDEV. ***p < 0.001. (**B**) (Left) Cells of the MCF10A progression series stained with superoxide probe. Scale bars: 20 μm. (Right) Quantification of superoxide signal/cell. (**C**) Total level of reactive oxygen species in the MCF10A progression series. (**D**) BH_4_ level of the MCF10A progression series. Error bars: mean ± STDEV. ***p < 0.001. (**E**) Images of western blots against endogenous NOS inhibitors, ADMA and DYNLL1, for cell lines of the MCF10A progression series. β-actin serves as the loading control. (See the raw gel image in Supplementary Fig. 3).

To test this possibility, we measured the intracellular BH_4_ level in cell lines of the breast cancer progression series. As expected, BH_4_ level was dramatically (~−40%) reduced in normal-to-precancerous transition and remained low thereafter (Fig. 2D). This paralleled the dramatic increase of superoxide levels, suggesting the possible occurrence of NOS uncoupling (Fig. 2B). We additionally tested for the potential involvement of known endogenous NOS inhibitors, asymmetric dimethylarginine (ADMA) and dynein light chain LC8-Type 1 (DYNLL1)^92^, in reduction of NO level in the progression series. ADMA and DYNLL1 levels did not significantly change throughout the progression series (except for some increase of ADMA in DCIS) (Fig. 3E, Supplementary Fig. 3), excluding their major contributions. These results altogether strongly suggest that deficiency of the NOS cofactor, BH_4_, is a critical contributor to reduction of basal NO producion in MECs during cancer progression.

### Pharmacological deprivation of NO impairs mouse mammary gland development, but induces desmoplastic ECM due to overactivation of TGFβ

**Figure 4:**
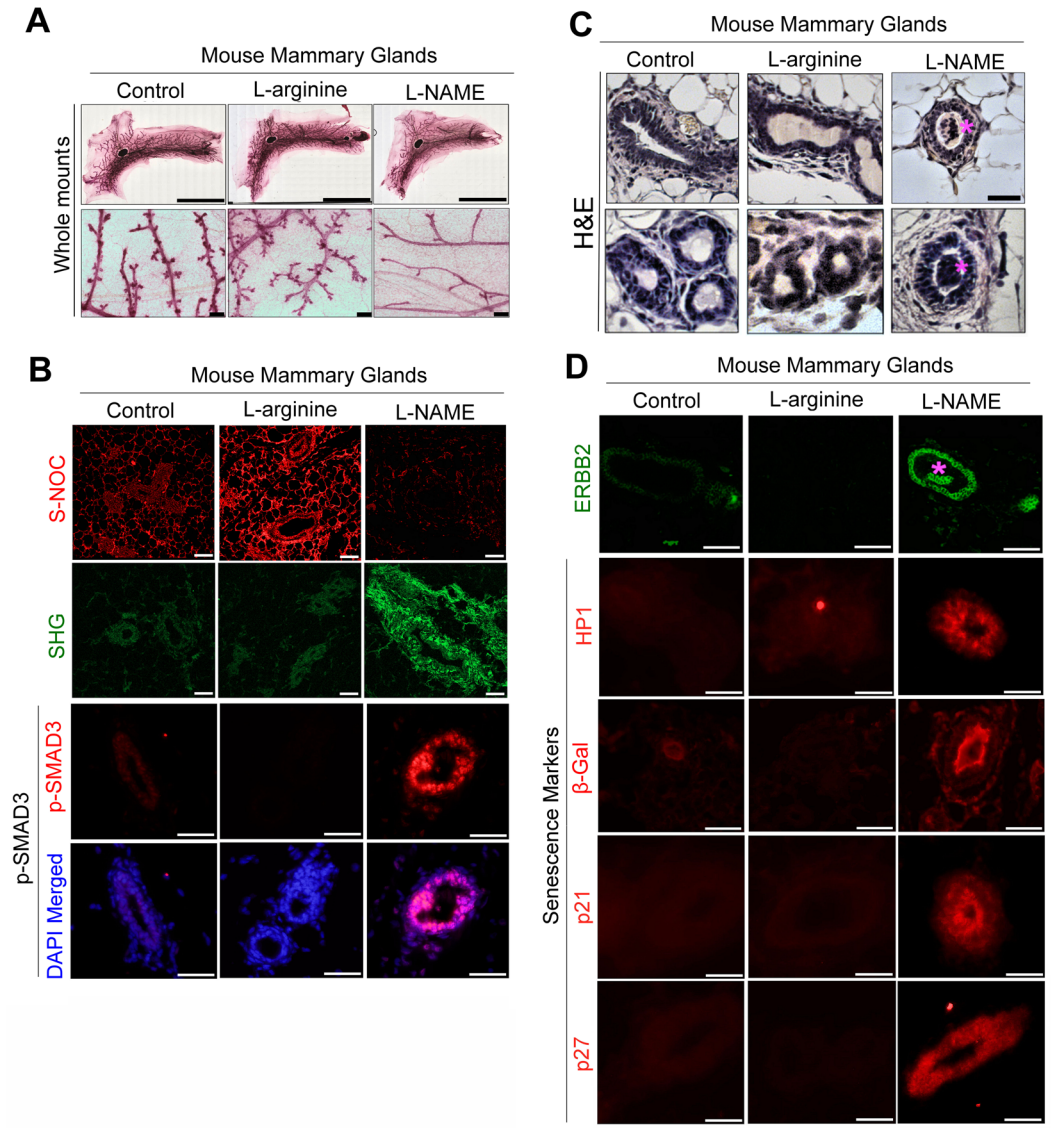
Deprivation of NO in developing mouse mammary glands stiffens the ECM and induces precancerous lesions, accompanied by upregulation of TGFβ and ERBB2 and induction of senescence. (**A**) Whole mounts of carmine-stained 4^th^ mammary glands of mice after i.p. treatment with control (PBS), L-arginine or L-NAME for 6 weeks (from 4 weeks to 10 weeks old). Scale bars: top, 10 mm; bottom, 200 μm. (**B**) Representative images of S-NOC staining (top), SHG (collagen) (second row) and phospho-SMAD3 (TGFβ signal) staining (bottom 2 rows) of drug-treated mammary glands. Nuclei were counterstained with DAPI (blue). Scale bars: 50 μm. (See the original micrographs and quantification of S-SNOC, SHG and phospho-SMAD3 levels in Supplementary Fig. 4A–C). (**C**) H&E staining of drug-treated mammary glands. Note that intraductal papillomas are indicated with pink asterisks (*). Scale bars: 50 μm. (**D**) Representative images of staining for ERBB2 (top), HP1 (second row), β-Gal (third row), p21 (fourth row) and p27 (bottom row) of drug-treated mammary glands. Nuclei were counterstained with DAPI (blue). Scale bars: 50 μm (See the quantification of ERBB2, HP1, β-Gal, p21 and p27 staining in Supplementary Fig. 4D–H).

To determine whether reduction of NO level contributes to breast cancer pathogenesis *in vivo*, we pharmacologically inhibited NO production in inbred wild-type mice (BALB/c) for a duration of 6 weeks (from 4 to 10 weeks old) by intraperitoneal (i.p.) injection of L-NAME (NOS antagonist, 20 mg/kg), in comparison to control (vehicle) and L-arginine (NOS substrate [agonist], 20 mg/kg) treatments. L-NAME-treated mouse mammary glands completely lacked alveoli, demonstrating the clear developmental defect, consistent with NO’s critical roles in mammary gland development (Fig. 4A)^27,29,31–34^. To confirm altered NO levels, drug-treated mammary glands were stained for SNOC. Control glands exhibited the basal level of SNOC, whereas L-arginine-treated glands showed dramatically elevated SNOC level. On the other hand, L-NAME-treated glands exhibited almost undetectable SNOC level (Fig. 4B, Supplementary Fig. 4A).

Reduced NO bioavailability in patients with chronic conditions, such as diabetes, cardiovascular disease and obesity, often leads to formation of stiff, desmoplastic (fibrotic) ECM which is directly linked to increased cancer risk^93–95^. To test this possibility, we measured the density of collagen fibers in drug-treated mammary tissues using second harmonic generation (SHG) technique^96^. As expected, periductal collagen level was dramatically elevated in L-NAME-treated mammary tissues, compared to control or L-arginine-treated tissues (Fig. 4B, Supplementary Fig. 4B). To determine the cause of the desmoplastic ECM in L-NAME-treated mammary tissues, we tested for the involvement of transforming growth factor β (TGFβ), the major activator of collagen biosynthesis^97^. We stained mammary tissues for phospho-SMAD3 (active form), the downstream effector of TGFβ signaling. Phospho-SMAD3 level, in fact, was dramatically (>5-fold) elevated in L-NAME-treated mammary glands (Fig. 4B, Supplementary Fig. 4C), suggesting strong activation of TGFβ signals upon NO depletion. These results demonstrate that NO deprivation in mammary glands of prepubertal to pubertal mice impairs gland development, but induces desmoplastic ECM along with overactivation of TGFβ signaling.

### NO-deprived mouse mammary glands form precancerous lesions that overexpress ERBB2

Formation of desmoplastic ECM indicates the occurrence of certain pathological conditions in NO-deprived mammary tissues^95^. Histological examination strikingly revealed that every L-NAME-treated animal (n = 6) formed multiple (peripheral) papillomas —precancerous mammary lesions^54–58^ —visualized by H&E staining of serial sections. In contrast, neither control nor L-arginine-treated animals formed such lesions (Fig. 4C). We then tested for the possible involvement of the ERBB2 oncogene, highly linked to precancerous progression of MECs^98–101^. While a change in the ERBB2 gene locus is found in 20~30% cases of invasive breast cancers, it is even more prevalent in premalignant breast lesions (~50%), suggesting that a change in ERBB2 gene is an early event in breast carcinogenesis^102–104^. As expected, ERBB2 levels were dramatically (>3-fold) elevated in L-NAME-treated mammary glands (Fig. 4D, Supplementary Fig. 4D). It is reported that overexpression of ERBB2 in normal or cancerous breast cells induces cellular senescence as an anti-carcinogenic mechanism^105–107^. In addition, senescence is shown to be the most prevalent in precancerous lesions, compared to normal or invasive lesions^59–61^. Therefore, to test whether the L-NAME-treated glands had undergone senescence, we determined the levels of well-established senescence markers: heterochromatin protein 1 (HP1), β-Galactosidase (β-Gal), p21 and p27^60,61,108^. All these senescence markers were highly elevated in L-NAME-treated glands, whereas they were almost undetectable in control or L-arginine-treated glands (Fig. 4D, Supplementary Fig. 2E–H). These results demonstrate that NO deprivation in mouse mammary glands induces precancerous lesions that strongly express ERBB2, accompanied by the occurrence of senescence.

### Inhibition of NO in non-malignant human mammary epithelial cells in 3D ECM cultures impairs acinar morphogenesis and upregulates TGFβ and ERBB2

**Figure 5:**
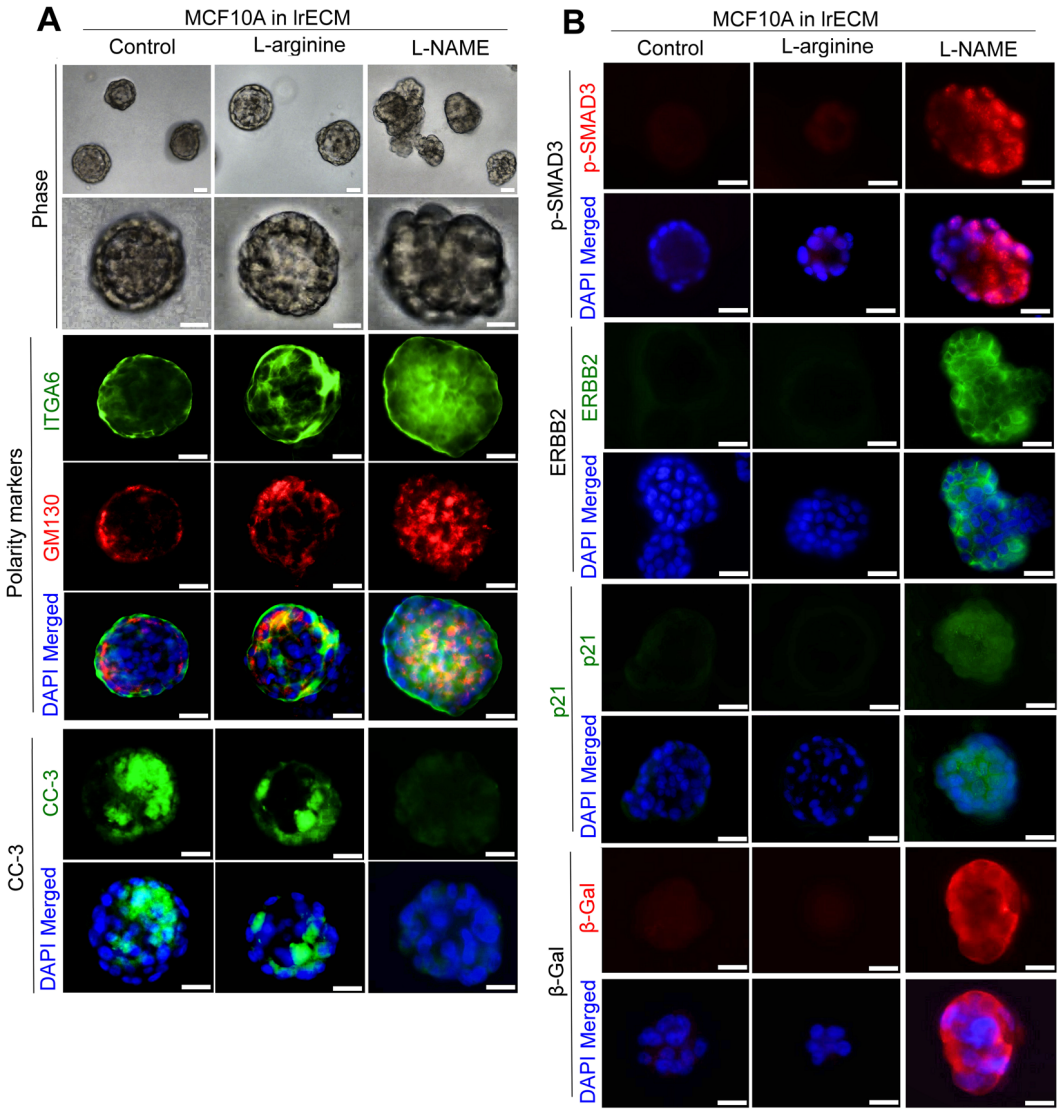
Deprivation of NO in non-malignant mammary epithelial cells in 3D cultures induces disorganized colonies, accompanied by induction of TGFβ, ERBB2 and senescence markers. (**A**) Representative images of MCF10A cells cultivated in 3D ECM under treatment of control (PBS), L-arginine or L-NAME for 3 weeks. Top 2 rows: phase images. Middle 3 rows: staining for the basal marker, integrin α6 (ITGA6) or apical marker, GM130. Bottom 2 rows: staining for lumen marker, cleaved caspase 3 (CC-3). Nuclei were counterstained with DAPI (blue). Scale bars: 20 μm. (See the quantification of colony size, and plot profiles of ITGA6, GM130 and CC-3 signals in Supplementary Fig. 5A,B). (**B**) Representative images of phospho-SMAD3 (top 2 rows), ERBB2 (rows 3–4), p21 (rows 5–6) or β-Gal (bottom 2 rows) staining of drug-treated MCF10A cells in 3D cultures. Nuclei were counterstained with DAPI (blue). Scale bars: 20 μm. (See the quantification in Supplementary Fig. 5C).

To determine whether the effects of NO modulation on mouse mammary glands were intrinsic to MECs or due to stromal and systemic influence, we treated mono-cultures of non-malignant human MECs (MCF10A cells) with L-NAME, in comparison to control (PBS) or L-arginine, in 3D lrECM for 3 weeks. Consistent with our previous report^24^, L-NAME impaired formation of mammary acini, but induced formation of disorganized, proliferative aggregates (Fig. 5A, Supplementary Fig. 5A). These colonies showed aberrant apico-basal polarity, indicated by mislocalization of the basal marker (integrin α6) and apical marker (GM130), as well as the loss of lumens (demarcated by cleaved caspase 3) (Fig. 5A, Supplementary Fig. 5B)^24^. L-NAME dramatically elevated phospho-SMAD3 and ERBB2, as well as senescence markers, p21 and β-Gal in MECs in 3D lrECM cultures (Fig. 5B, Supplementary Fig. 5C)^109^, consistent with the above *in vivo* results (Fig. 4B,D).

To determine the generality of the effects of modulating NO level, we applied L-arginine or L-NAME to ERBB2-positive (amplified) SKBR3 breast cancer cells^110^. Surprisingly, even after a short-term (overnight) treatment, L-NAME dramatically (>3-fold) elevated the level and membrane-localization of ERBB2 over control, whereas L-arginine almost abrogated them (Supplementary Fig. 5D). These results suggest that the effects of NO modulation on mammary glands we observed are largely intrinsic to MECs and might have manifested soon after treatment.

### Inhibition of NO in breast epithelial cells induces bi-lineage and stem cell-like phenotype in both mammary glands and 3D ECM cultures

It has been reported that activation of certain oncogenic pathways, such as ERBB2, in normal or cancerous MECs could induce stem cell-like properties^101,111^. These cells are characterized by luminal (CK8/18)/basal (CK14) bi-lineage phenotype, as well as high expression of stem cell markers such as CD44, and are also found to be prevalent in precancerous lesions^62–64^.

**Figure 6:**
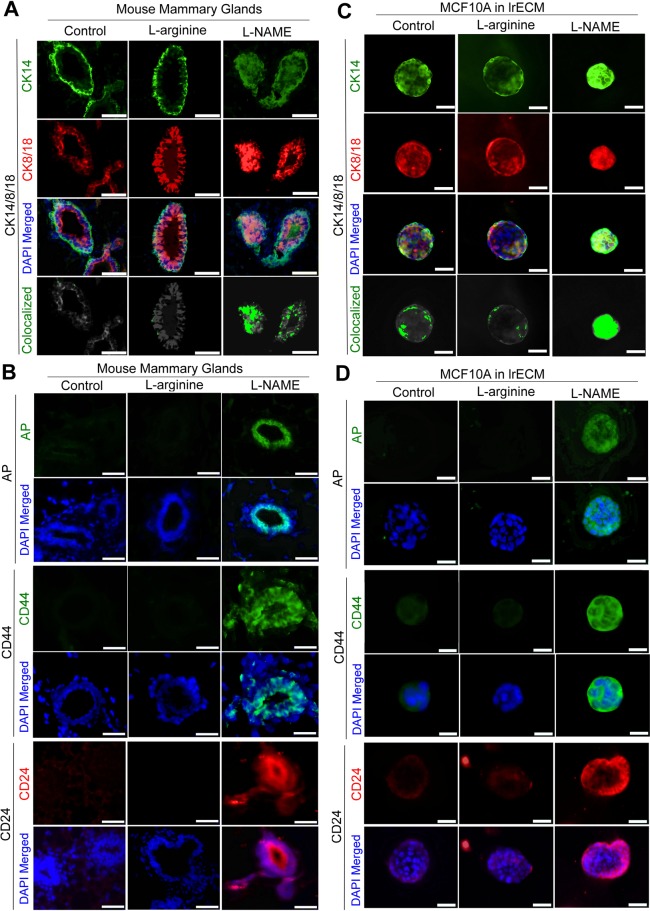
Deprivation of NO induces bi-lineage phenotype and expression of stem cell markers in mammary epithelial cells both *in vivo* and in 3D cultures. (**A**) CK14 (basal) and CK8/18 (luminal) staining of mouse mammary glands treated with control (PBS), L-arginine or L-NAME for 6 weeks. Co-localization of CK14 and CK8/18 was captured with ImageJ. Scale bars: 100 μm. (See the quantification in Supplementary Fig. 6A). (**B**) Representative images of drug-treated mammary glands stained for stem cell markers: alkaline phosphatase (AP) (top 2 rows), CD44 (middle 2 rows) or CD24 (bottom 2 rows). Nuclei were counterstained with DAPI (blue). Scale bars: 50 μm. (See the quantification in Supplementary Fig. 6B–D). (**C**) CK14 and CK8/18 staining of non-malignant MCF10A cells cultivated in 3D ECM in the presence of control (PBS), L-arginine or L-NAME for 3 weeks. Scale bars: 50 μm. (See the quantification in Supplementary Fig. 6E). (**D**) Drug-treated MCF10A cells in 3D cultures stained for AP (top 2 rows), CD44 (middle 2 rows) or CD24 (bottom 2 rows). Nuclei were counterstained with DAPI (blue). Scale bars: 50 μm. (See the quantification in Supplementary Fig. 6F–H).

Since NO deprivation dramatically elevated ERBB2 in wild-type mouse mammary glands (Fig. 4D, Supplementary Fig. 4D), we tested whether this might have induced stem cell-like properties^101^. First we tested for the occurrence of bi-lineage phenotype by co-staining drug-treated mammary glands for CK8/18 (luminal) and CK14 (basal)^62^. As previously reported, control and L-arginine-treated mammary glands showed mutually exclusive pattern of CK8/18 vs. CK14^112^. In contrast, L-NAME-treated glands showed a significant (>10-fold) increase in CK18^high^/CK14^high^ bi-lineage cells (Fig. 6A, Supplementary Fig. 6A)^62^.

Next, we tested for the induction of stemness by staining the drug-treated mammary glands for stem cell markers, alkaline phosphatase (AP, a pluripotent stem cell marker)^113^, CD44 (another stem/progenitor marker) and CD24 (the luminal lineage marker)^114–116^. In both humans and mice, CD44^high^/CD24^low^ phenotype is the predominant marker for multipotent stem/progenitor cells that have the highest repopulating (i.e., tumor-initiating) potential, while CD44^high^/CD24^high^ and CD44^low^/CD24^high^ phenotypes are the markers for cells “*committed*” to luminal differentiationin^114–117^. (CD44^high^/CD24^high^ phenotype, nevertheless, is linked to increased drug-resistance^118^). In control and L-arginine-treated glands, the three stem cell markers, AP, CD44 and CD24, were virtually all absent. In contrast, in L-NAME-treated mammary glands, both AP and CD44 were strongly expressed in all the epithelia, whereas CD24 was mostly expressed in the luminal layer and expressed at low to moderate levels in the basal layer. (Fig. 6B. Supplementary Fig. 6B–D). These results demonstrate that L-NAME-treatment of mammary glands induces the stem cell population (CD44^high^/CD24^low^) on the basal layer, as well as another population of cells committed to luminal differentiation (CD44^high^/CD24^high^) on the luminal layer^117^.

We tested whether this *in vivo* phenotype could be recapitulated in mono-cultures of human MECs in 3D lrECM. Similar to our *in vivo* results, 3D colonies of control and L-arginine-treated non-malignant MCF10A cells showed distinct bi-layers of CK8/18-positive (luminal) vs. CK14-positive (basal) cells (Only 5~7% of cells were CK18^high^/CK14^high^ bi-lineage). In contrast, L-NAME-treated cells were virtually all (>70%) CK18^high^/CK14^high^ bi-lineage (Fig. 6C, Supplementary Fig. 6E). Stem cell markers, AP, CD44 and CD24, were almost undetectable in control and L-arginine-treated colonies. Conversely, in L-NAME-treated colonies, the expression of AP and CD44 was strongly positive throughout; CD24 expression was highly elevated on the periphery, but remained low in the center (marking the peripheral cells as CD44^high^/CD24^high^ vs. central cells as CD44^high^/CD24^low^) (Fig. 6, Supplementary Fig. 6F–H). These results altogether demonstrate that L-NAME-treatment of normal/non-malignant MECs of humans and mice, both in culture and *in vivo*, respectively, induces a stem cell-like population with CK18^high^/CK14^high^ bi-lineage phenotype and CD44^high^/CD24^low^ expression.

### Inhibition of NO in breast epithelial cells promotes formation of mammospheres enriched for stem cell-like cells

**Figure 7:**
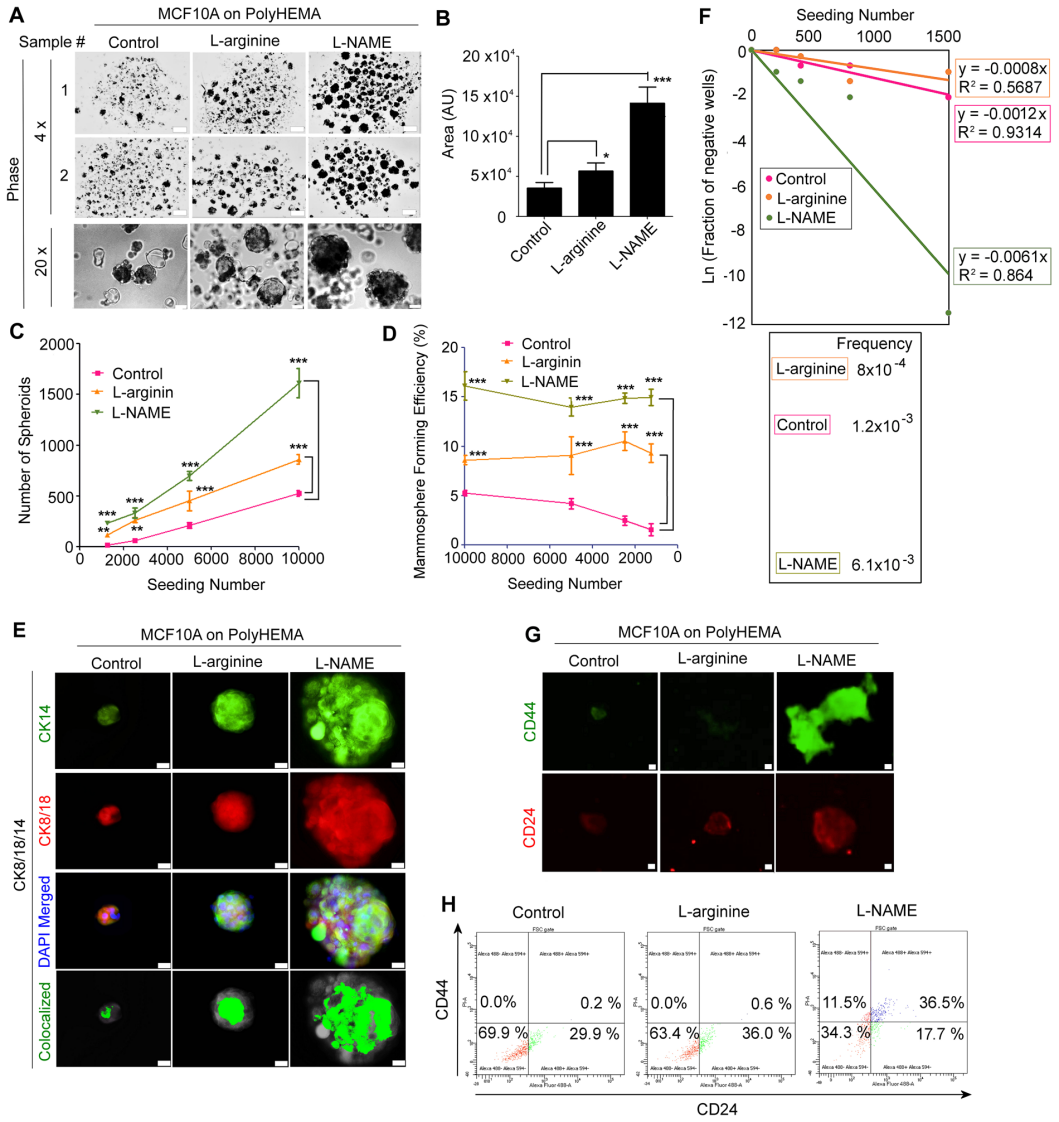
Deprivation of NO elevates self-renewal potential of non-malignant mammary epithelial cells. (**A**) Representative images of mammospheres derived from MCF10A cells grown on PolyHEMA-coated plates under the treatment of control (PBS), L-arginine or L-NAME for one week. Scale bars: 200 μm (top 2 rows) and 50 μm (bottom row). (**B**) The size of mammospheres derived from drug-treated MCF10A cells. (**C**) The number of spheroids formed at each seeding number. (See the micrographs of spheroids in Supplementary Fig. 7A). (**D**) Mammosphere forming efficiency at each seeding number. Mammosphere forming efficiency (%) = (# of spheroids)/(# of cells seeded) × 100^119,120^. Statistical significance between different treatments was determined by two-way ANOVA analysis with Bonferroni post hoc test^175^. Error bars: mean ± STDEV. **p < 0.01; ***p < 0.001. (**E**) Staining of mammospheres for the basal marker, CK14, and luminal marker, CK8/18. Co-localization of CK14 and CK8/18 was captured with ImageJ. (See the quantification in Supplementary Fig. 7B). (**F**) Limiting dilution analysis to determine the sphere forming frequency at clonal densities (≥200 cells/96-well)^126^. (Top) The graph plotted with the Y-axis as Ln (fraction of negative wells) and X-axis as seeding number. (Bottom) The sphere forming frequency corresponding to −(slope) of the line of best fit. (**G**) Staining of paraffin-embedded/sectioned mammospheres^129^ for stem cell markers CD44 and CD24. Scale bars: 20 μm (see the quantification of CD44 and CD24 levels in Supplementary Fig. 7C,D). (**H**) FACS analysis to determine the expression of CD44 and CD24 in cells dissociated from mammospheres. Isotype controls were used to set threshold gate.

To further validate the induction of stemness by L-NAME treatment, drug-treated MCF10A cells were cultivated on PolyHEMA-coated (non-adherent) plates, which allows for detection of self-renewal capacity through formation of mammospheres^119–124^. For mammosphere formation assay as described previously^119,120,123,124^, cells were seeded at the densities of 1,250~10,000 cells/48-well. Both L-NAME and L-arginine-treatments increased the size, number and formation efficiency of mammospheres over control; however, L-NAME yielded the values twice as much as L-arginine (Fig. 7A–D, Supplementary Fig. 7A). The proportions of CK18^high^/CK14^high^ bi-lineage cells within mammospheres were significantly (>4-fold) higher in L-NAME and L-arginine-treated spheroids than control spheroids (Fig. 7E, Supplementary Fig. 7B), attesting to the increase of stem cell-like populations^62,112,125^.

Aiming to differentiate the effects of L-NAME from those of L-arginine, we performed a limiting dilution assay on drug-treated MCF10A cells that determines the abundance of cells capable of forming spheroids at clonal densities (200~1600/96-well)^126,127^. L-NAME treatment elevated the abundance of spheroid-forming cells by 5-fold over control and 8-fold over L-arginine treatment (Fig. 7F). These results altogether demonstrate that L-NAME treatment greatly increased the abundance of stem cell-like cells that possess clonal expansion capacity.

To further confirm the stemness of drug-treated spheroids, we measured the levels of stem cell markers, CD44 and CD24^128^, by immunofluorescence imaging of paraffin-embedded/sectioned spheres^129^. L-NAME-treated spheroids were strongly positive for CD44, whereas L-arginine-treated spheroids showed little or no expression. CD24 expression, on the other hand, was low in all conditions (Fig. 7G, Supplementary Fig. 7C,D).

As a complementary approach, we performed FACS analysis on cells dissociated from mammospheres. Consistent with the result from immunofluorescence imaging, L-NAME-treated mammospheres showed a dramatic increase in CD44 level. About 50% of cells were CD44 positive, and 1/4 of these CD44-positive cells (11.5% of the total cells) were CD44^high^/CD24^low^, cells with the highest repopulating potential^114–117^ (Fig. 7H). On the other hand, there were almost no (0.2~0.6%) CD44-positive cells in control and L-arginine-treated mammospheres. While CD24 level increased in L-arginine-treated (+6.5%) and L-NAME-treated spheroids (+24.1%), a significant fraction of cells (46~65%) showed low expression levels (Fig. 7H). These results altogether demonstrate that L-NAME-treatment elevated stem cell-like cells (CD44^high^/CD24^low^) which have the highest self-renewal ability and clonogenicity^128^.

### Normalization of NO levels with the BH_4_ precursor, sepiapterin, ameliorates the malignant phenotype of breast cancer cells

To further test whether dysregulated NO levels contribute to the phenotype associated with breast cancer progression, we sought to normalize NO levels in precancerous and cancerous MECs. We showed above that reduced basal NO production in cultured MECs along with cancer progression was linked to oxidative depletion of the NOS cofactor, BH_4_, which triggers NOS uncoupling (Figs 1A–C, 2B–D)^49,88^. In an attempt to ameliorate the conditions of diseases including breast cancer, the BH_4_ precursor, sepiapterin, has been successfully utilized for *‘recoupling’* NOS in a number of cell culture and preclinical studies^49,130–134^.

**Figure 8:**
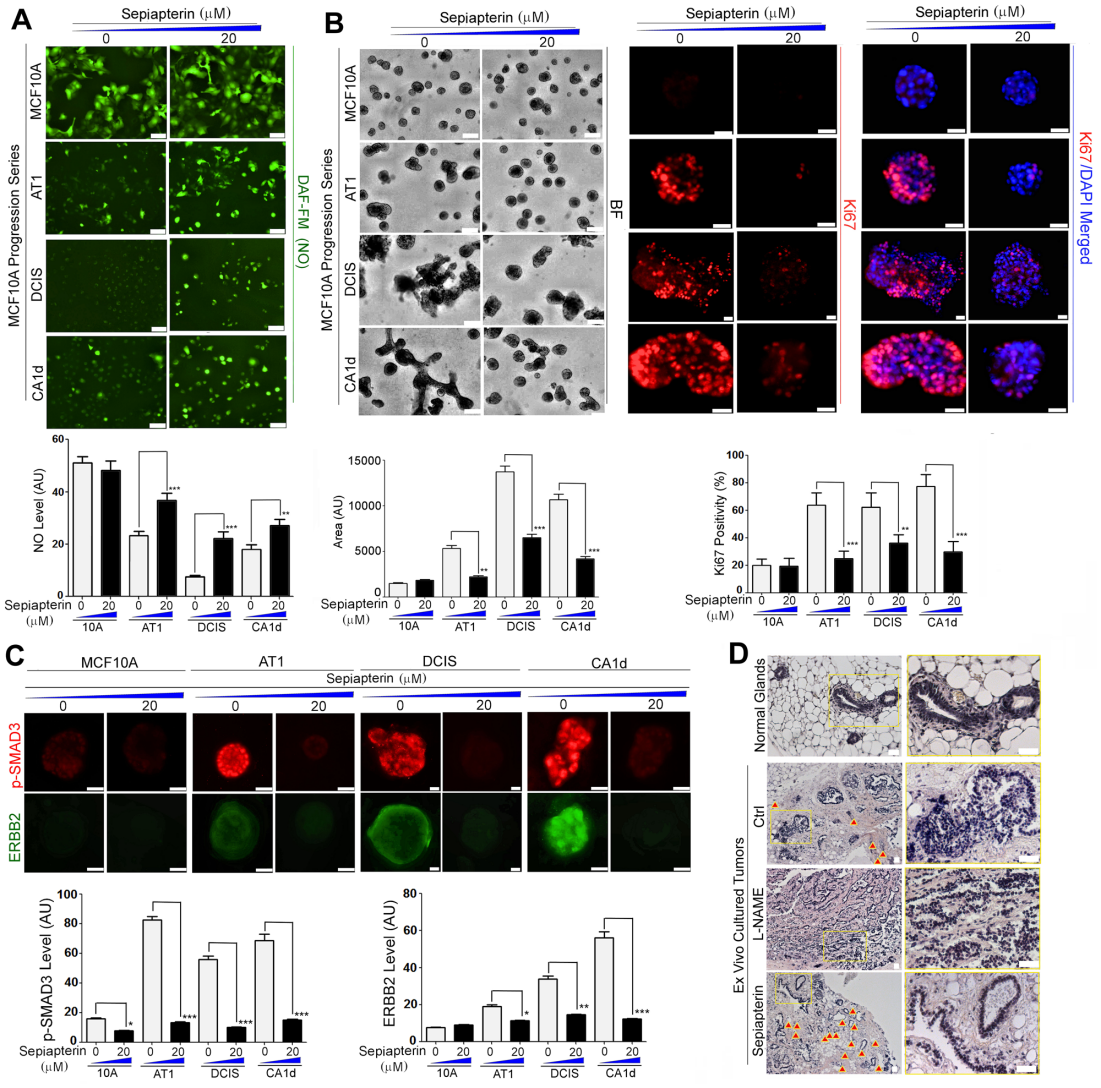
BH_4_ precursor, sepiapterin, normalizes NO level and ameliorates malignant phenotype of precancerous and cancerous cells. (**A**) (Top) Cell lines of the MCF10A progression series cultured in 5% Matrigel drip with or without sepiapterin (20 μM) and stained with the NO probe DAF-FM DA, Scale bars: 50 μm. (Bottom) Quantification of DAF-FM signal/cell. AU: Arbitrary unit. (**B**) (Left top) Representative images of the MCF10A progression series cultivated in 3D ECM with or without sepiapterin (20 μM). (Left bottom) Quantification of colony size. (Middle top) Cell lines in the series cultivated in 3D ECM with or without sepiapterin (20 μM) and stained for Ki67. Scale bars: 50 μm. (Right top) Nuclei were counterstained with DAPI (blue) and merged with Ki67. Scale bars: 50 μm. (Right bottom) Percent of Ki67-positive cells/colony. (**C**) (Top) Representative images of the progression series cultivated in 3D ECM with or without sepiapterin (20 μM) and stained for phospho-SMAD3 or ERBB2. Nuclei were counterstained with DAPI (blue). Scale bars: 20 μm. (Bottom) Quantification of phospho-SMAD3 (left) and ERBB2 levels (right) of the progression series. Error bars: mean ± STDEV. *p < 0.05; **p < 0.01 and ***p < 0.001. Note that the effects of sepiapterin are only discernible in precancerous and cancerous cells, but not in non-malignant cells. (See the results of different concentrations of sepiapterin in Supplementary Fig. 8). (**D**) Mammary tumors from MMTV-PyMT mice (18 weeks, n = 4) were *ex vivo* 3D cultured with vehicle (PBS), L-NAME (2.5 mM, NOS inhibitor) or sepiapterin (100 µM) for 1 week. First raw: normal mouse mammary glands. Left column: 100x; right column: 400x H&E images of the boxed areas of the images in left column. Arrow heads: vessels. Scale bars: 50 µm. Note the restoration of normal-like glands after sepiapterin treatment vs. worsened proliferative phenotype after L-NAME treatment. Also, note the significant increase in the vasculature after sepiapterin treatment.

We tested whether the application of sepiapterin could help normalize NO levels in precancerous and cancerous MECs in 3D lrECM cultures and whether this could ameliorate the malignant phenotype. After cultivation of the MCF10A progression series in the presence of sepiapterin at 20 μM^49^, NO levels of precancerous and cancerous cell lines (AT1, DCIS and CA1d) were restored to the levels comparable to (or at least half of) the level of non-malignant MCF10A cells. In contrast, NO level in non-malignant MCF10A cells did not change after sepiapterin treatment (Fig. 8A). Importantly, 20 μM of sepiapterin dramatically (>50%) reduced proliferation indices (i.e., colony size and Ki67 positivity) of precancerous and cancerous cell lines, but not of non-malignant cells (Fig. 8B). This was accompanied by a dramatic reduction in the levels of phospho-SMAD3 and ERBB2, which otherwise were pronounced in precancerous and cancerous cells (Fig. 8C). These results confirm that deficiency of BH_4_ is a major contributor to reduced basal NO production and malignant phenotype of precancerous and cancerous MECs.

To test whether these effects of sepiapterin are dose-dependent, we treated the MCF10A progression series cultivated in 3D lrECM with a different concentration of sepiapterin (0, 20 or 100 μM). The increasing concentrations of sepiapterin (20 to 100 μM) progressively normalized NO levels in precancerous and cancerous cells, without affecting that of MCF10A cells (Supplementary Fig. 8A). Nevertheless, the growth-suppressive effects (by colony size and Ki67 positivity) of sepiapterin did not differ between the concentrations of 20 and 100 μM (Supplementary Fig. 8B–D). This suggests that the activity of sepiapterin is threshold-dependent, rather than concentration-dependent, at least within this range.

Furthermore, sepiapterin (20 and 100 μM) helped “normalize” the cellular phenotype of precancerous and cancerous cells of the MCF10A progression series also in a threshold-dependent manner. In particular, AT1 cells restored the apico-basal polarity, indicated by the proper localization of integrin α6 (the basal marker) and cleaved caspase 3 (a marker of lumens)^24,135^ analogous to non-malignant MCF10A cells (Supplementary Fig. 9A,B). In DCIS and CA1d cells, apico-basal polarity was only partially restored by sepiapterin treatment (Supplementary Fig. 9A,B). Nevertheless, the expression of CK14 (basal cell marker) and the central localization of CK8/18 (luminal cell marker) were almost completely restored (Supplementary Fig. 9C).

We applied sepiapterin to *ex vivo* 3D cultured mammary tumors from MMTV-PyMT mice (18 weeks old) and tested for its anti-tumor effects. Sepiapterin greatly reversed the proliferative phenotype of the epithelia and even partially restored normal-like glands within tumors in a week. Nevertheless, it needs to be noted that sepiapterin also significantly elevated the vascular density in tumors in support of a notion that the increase of NO promotes antiogenesis^20,136,137^ (Fig. 8D).

To test the generality of the effects of sepiapterin on breast cancer cells, we pulse-treated different breast cancer cell lines (basal type: MDA-MB231 and MDA-MB468; luminal type: MCF7 and SKBR3) with a different concentration of sepiapterin (0, 20, or 100 μM) in lrECM cultures. Even after two hours of treatment, both 20 and 100 μM of sepiapterin equally increased NO levels of these cancer cell lines by 2~3-fold (Supplementary Fig. 10A). Surprisingly, sepiapterin decreased Ki67 positivity of cancer cells by 60~100% (luminal cells showed better responsiveness than basal cells) (Supplementary Fig. 10B). This result augments a previous report of the growth-inhibitory effects of sepiapterin on breast cancer cells in culture and in animals after 2 days of treatment^49^.

As a complementary approach, we administered a low concentration (2.5 μM) of a NO donor, SNAP or GSNO, to another breast cancer progression series, HMT-3522 S1 (non-malignant) and T4-2 cells (malignant)^24,68,69^, cultivated in 3D ECM. This concentration of NO donors were chosen in an attempt to induce production of the physiological level (1–4 μM) of NO previously reported by us and others^24,138,139^. Similar to sepiapterin, NO donors at 2.5 μM significantly inhibited growth of tumor cells and restored their apico-basal polarity to the level analogous to that of non-malignant cells (Supplementary Fig. 11A–C). To test whether these effects of NO donors are dose-dependent, we administered a different concentration of SNAP or GSNO (0, 2.5 or 10 μM) to the HMT-3522 progression series cultivated in 3D lrECM. Interestingly, while the lower level (2.5 μM) of SNAP and GSNO restored the S1 cell-like polarity and growth-arrested phenotype in T4-2 cells, the higher level (10 μM) of the same NO donors showed no such effects (Supplementary Fig. 12A–C). This result is in line with the well-documented concentration dependence of NO’s bioactivities in cancer^17–20^.

The findings altogether suggest that normalization of basal NO level may have a therapeutic potential for breast cancer.

## Discussion

Advances in imaging and screening technologies have enabled the detection of precancerous, early-stage breast lesions, the majority of which are ductal carcinoma *in situ* (DCIS)^2^. Precancerous lesions arise from clonal expansion of a single cell^140^. Precancerous lesions are the precursors to invasive breast cancers, and about 40% of them could progress to invasive forms, if untreated^2^. It is, however, not fully understood what drives formation of precancerous breast lesions, making the management of the disease challenging^2^.

In the study, we hypothesized that aberrant NO production might contribute to the formation of precancerous breast lesions and tested this possibility. Utilizing the MCF10A breast cancer progression series, we showed that the basal NO level in MECs plummeted along with breast cancer progression, consistent with our previous finding using another breast cancer progression series, HMT-3522^24^. In particular, such decline was the most notable in non-malignant to precancerous transition (Fig. 1A). Reduction of basal level of NO in MECs along with cancer progression was largely attributed to reduction of the NOS cofactor BH_4_ under increased oxidative stress (Fig. 3B–D). In contrast, the levels of NOS-1 and -3, the enzymes responsible for production of the basal level of NO in the breast, remained unchanged during cancer progression and, thus, were irrelevant to reduction of NO (Fig. 2A).

To confirm the pathological relevance of reduced basal NO production to breast carcinogenesis, we pharmacologically inhibited NO production in prepubertal to pubertal mice using L-NAME, an unreactive L-arginine analog. Previous studies report somewhat conflicting observations for the effects of L-NAME on cancer. Some studies report anti-cancer effects of L-NAME on already established cancer cells^141–143^, while others report the opposite^144–147^. Such discrepancy may reflect the complex activities of NO and NOS in cancer^18,20,24,35–43,47–50,145,148,149^ as well as possible co-suppression of NOS and arginase by L-NAME at high concentrations (>5 mM)^84,85,150^. L-NAME’s effect on the pathophysiology of normal cells, on the other hand, has yet to be explored.

L-NAME treatment of developing mouse mammary glands impaired alveologenesis, inducing precancerous lesions and desmoplastic ECM (Fig. 4A–C). Consistently, these mammary glands showed overactivation of ERBB2, closely linked to formation of precancerous lesions^100,101^, and fibrogenic TGFβ (Fig. 4B,D)^97^. Activation of these pathways were accompanied by the induction of senescence and stem cell-like properties, both prevalent in precancerous lesions (Figs 4D, 6B)^59,62,63,101^. We further showed that normalization of NO levels, by the use of the BH_4_ precursor (sepiapterin) or the physiological level of NO donor (SNAP or GSNO), suppressed TGFβ and ERBB2 signals and ameliorated the malignant phenotype of precancerous and cancerous MECs (Fig. 8A–D, Supplementary Fig. 11A–C).

NO’s bioactivities are largely dependent on its concentration, timing and context^17–19^. In healthy tissues, NO production is tightly regulated to attain the right condition^151^. In disease states, on the other hand, NO production is often dysregulated, leading to a deficient or excessive level of NO that, in either case, could contribute to the disease pathogenesis^39,40^. Such a complex, paradoxical role of NO in disease conditions, especially in cancer, have led to conflicting reports and a notion that NO plays a double-edged role as both a cancer-promoter and -inhibitor^17,18,20^. The enigma of NO’s role in cancer might be partly resolved by clarifying how NO is involved in a specific stage of a particular type of cancer under a certain context.

The present study demonstrates that maintenance of the physiological NO level in the breast exerts protective effects against formation of precancerous lesions. NO production, however, could be impaired by oxidative stress that depletes the NOS cofactor BH_4_, contributing to pathogenesis. Under such a condition, NOS is dysfunctional (uncoupled) and produces superoxide instead of NO, exacerbating oxidative stress^49,88^. In line with this notion, we observed that NO and BH_4_ levels both dramatically declined along with an increase in oxidative stress in precancerous and cancerous MECs cultivated in 3D lrECM (Figs 1A and 3B–D).

One of the most compelling findings in this study is that NO deprivation simultaneously upregulated TGFβ and ERBB2 in MECs both *in vivo* and *in vitro* (Figs 4B,D and 5A,B). TGFβ and ERBB2 are both under tight regulation in healthy tissues, whereas they become dysregulated during carcinogenesis^152,153^. TGFβ activity is primarily regulated post-translationally, especially through formation of the latent complex along with LTBP1 and LAP^154^. ERBB2 elevation, in contrast, is primarily ascribed to gene amplification^155^. However, other mechanisms that do not involve changes in DNA sequence also play major roles in regulation of ERBB2 level. These mechanisms include transcriptional and post-transcriptional activation^156–158^ as well as protein stabilization^159^. In fact, it is shown that ~20% of ERBB2-positive tumors detected by IHC (score of 3+) do not harbor amplification of the ERBB2 gene^160,161^. We are postulating that the basal steady-state level of NO produced in the healthy breast tissue might negatively regulate proteins involved in upregulation of TGFβ and ERBB2 via S-nitrosylation (SNO). SNO plays a large part of NO signal, regulating structures and functions of >3000 proteins^162^. In healthy cells, a subset of proteins are constitutively S-nitrosylated at the basal level to remain under control^25^. In disease states including cancer, on the other hand, SNO level could be dysregulated, contributing to disruption of homeostasis^25^. Currently, we are examining the potential role of SNO in suppressing TGFβ and ERBB2 expression and activity in non-malignant MECs.

Our study demonstrates that normalization of NO level with the BH_4_ precursor, sepiapterin (20 or 100 μM), or with a low level (2.5 μM) of a NO donor, SNAP or GSNO, reduced ERBB2 and TGFβ signals and suppressed the proliferative phenotype of precancerous and cancerous MECs in 3D lrECM. NO normalization also restored cell polarity and lineage markers in these cells (a process termed *‘phenotypic reversion’*^24^) (Fig. 8, Supplementary Figs 8–12). Surprisingly, application of sepiapterin to *ex vivo* 3D cultured mammary tumors partially restored normal-like glands and largely reversed the proliferative phenotype of tumor epithelia in a week (Fig. 8D). However, it is critical to note that modulation of NO levels should aim at restoring the physiological basal level of NO (1–4 μM) in MECs^24,138,139^. Too much or too little NO supplementation, on the other hand, could result in pathogenic effects because of the concentration-dependent NO’s bioactivities^18,20^. In fact, while NO donors at the lower concentration (2.5 μM) exerted anti-cancer activity, the same donors at the higher concentration (10 μM) showed no such activity (Supplementary Fig. 12A–C). In contrast, sepiapterin exerted the anti-cancer activity equally at both 20 and 100 μM, and thus was threshold-dependent at least within this range. These results suggest that sepiapterin might have a wider therapeutic window than NO donors.

Our present findings strongly suggest the translational potentials of sepiapterin (and possibly the physiological concentrations of NO donors) for treatment of early-stage breast cancer. However, the advancement of such approach would depend on the development of tumor-restricted drug delivery systems, because systemic administration of these agents could have potential drawbacks^17,163–165^. While systemic administration of sepiapterin to animals is shown to reduce tumor cell proliferation^49^, it also enhances vascularization of tumor tissues^20,136,137^. Consistently, when we applied sepiapterin to *ex vivo* 3D cultured mouse mammary tumors, their proliferative phenotype was dramatically suppressed, whereas the vasculature was significantly enhanced (Fig. 8D). Such enhanced vasculature by sepiapterin treatment might eventually help tumor cells re-grow and reduce the efficacy of the drug. To circumvent these drawbacks, we are currently developing liposome-based systems that could specifically deliver sepiapterin to the breast epithelia of lesions via specific homing peptides^166^. Moreover, future studies could possibly determine whether certain diet and exercise routines help maintain the physiological NO level in the breast and prevent formation of precancerous lesions.

## Materials and Methods

### Cell lines

Cell lines of the MCF10A breast cancer progression series were obtained from Karmanos Cancer Institute (MI, USA)^78^ under Material Transfer Agreement. Cell lines of the HMT-3522 breast cancer progression series (S1 and T4-2) were provided by Mina Bissell laboratory in Lawrence Berkeley National Laboratory (Berkeley, CA)^24^ under Material Transfer Agreement. All the other breast cancer cell lines were obtained from American Tissue Culture Collection (ATCC). All the cell lines had been authenticated by the providers through genome sequencing and STR profiling. Mycoplasma testing of these cell lines was negative.

### Cell culture and reagents

The MCF10A breast cancer progression series comprising non-malignant MCF10A, premalignant AT1, *in situ* carcinoma DCIS.COM and metastatic CA1d, was maintained as described^78^. The isogenic cell lines of the HMT-3522 human breast cancer progression series, comprising non-malignant S1 and malignant T4-2 cells, were maintained as described previously^24^. All the other breast cancer cell lines were maintained in DMEM/F12 with 10% FBS and 1% penicillin/streptomycin. For 3D culture experiments, cells were seeded at the density of 2.5 × 10^4^ cells/cm^2^ for non-malignant cells and 1.8 × 10^4^ cells/cm^2^ for malignant cells in growth factor reduced Matrigel (BD Biosciences) and maintained for 10–21 days with addition of fresh medium on alternate days. For inhibition of NO production, cells were treated with 2.5 mM L-NAME (N_ω_-Nitro-L-arginine methyl ester hydrochloride, Sigma- Aldrich); for induction of NO production, 2.5 or 10 μM SNAP (S-Nitroso-N-acetyl-DL-penicillamine,) or 2.5 or 10 μM GSNO (S-nitrosoglutathione, Sigma-Aldrich) was used. To compensate for the reduced BH_4_ level in cancer cells, 20 or 100 μM L-sepiapterin (BH_4_ precursor, Sigma-Aldrich) was used. The NOS inhibitors were all obtained from Cayman Chemical. NOS1 inhibitor (Nω-Propyl-L-arginine hydrochloride) was used at 10 µM; NOS2 inhibitor (1400 W) at 50 µM; and NOS3 inhibitor (L-NIO) at 10 µM^167^.

### Antibodies

The following antibodies were used. Anti-β-Galactosidase (Bioss, bs-4960R), anti-alkaline phosphatase (Novus Biologicals, NBP1-32948); anti-heterochromatin protein 1-γ (HP1-γ, phospho Ser 93, Bioss, bs-3221R); anti-p21 (Bioss, bs-10129R); anti-p27 (phospho Thr 187, Abcam, ab75908); anti-NOS-1, 2 (Thermo Fisher Scientific, PA1-033 and -036); anti-NOS-3 (Sigma-Aldrich, SAB-4300435); anti-ErbB2 (Thermo Fisher Scientific, PA5-16395); anti-pSMAD3 (Ser423/Ser425, Novous Biological, NBP1-77836); anti-CD24 (Novus Biologicals, NBP1-4639055); anti-CD44 (Bioss, bs-2507R); anti-S-Nitroso-Cysteine (Abcam, ab50185 or Alpha Diagnostics, NISC11-A); anti-Integrin α6 (BD Biosciences, 555734); anti-GM130 (Cell Signaling, 12480 S); anti-human CK 14 (ThermoFisher, MA511599); anti-human CK 18 (ThermoFisher, PA514263); anti-mouse CK 14 (BioLegend, 905301); anti-human CK 8/18 (DSHB, Troma-I); anti-Cleaved Caspase3 (Cell Signaling, #9664); anti-β-Actin (Sigma, A1978); anti-DYNLL1 (Abcam, ab51603); and anti-ADMA (EMD Millipore, 09-814).

### NO measurement in live cells

To capture the snap shot of NO production in live cells after Matrigel addition, a dye DAF-FM DA (4-Amino-5-Methylamino-2′,7′-Difluorofluorescein Diacetate, Life Technologies) was used according to the manufacturer’s protocol. Briefly, cells were seeded at 1.0 × 10^5^ cells/24 well-plate maintained in phenol red free growth media overnight and pre-treated with the dye for 1 h. After the drip (5%) of Matrigel (phenol red-free) was added, cells were incubated in dark for 1 h and washed in fresh media. Micrographs were taken on live cells by fluorescent microscope with FITC filter, and the signal intensity/cell was measured with ImageJ.

### Nitrite measurement

To quantify the cumulative level of nitric oxide produced by cells, more stable nitric oxide metabolite, nitrite, was measured based on the reaction of a dye DAN (diaminonaphthalene) by using Measure-IT High-Sensitivity Nitrite Assay Kit (Life technologies), according to the manufacturer’s protocol. Briefly, cells were plated at 1 × 10^6^ cells/60 mm-plate and maintained overnight. Cells were maintained in 2 ml of the fresh phenol red-free medium containing 5% Matrigel (phenol red-free, Corning, #356237) for 24 h. The conditioned medium was harvested and spun to remove the Matrigel. Ten μl of the cleared conditioned medium was reacted with the assay reagents in dark, and the signal intensity was measured using nitrite standards at the excitation/emission maxima of 340/410 nm.

### pH measurement

The pH of cell lines of the MCF10A progression series were measured with the pHrodo® Green AM Intracellular pH Indicator (Cat# P35373, Thermo Fisher Scientific) according to the manufacturer’s protocol. Briefly, live cells were incubated with the pH probe for 30 min. For standard samples, the standard solution (Cat# P35379, Thermo Fisher Scientific) for each different pH was added at the end of 30 min, and cells were incubated for another 5 min. Cells were washed in fresh media, and micrographs were taken on live cells by fluorescent microscope with FITC filter. The signal intensity per cell was quantified by ImageJ.

### Measurement of oxidative stress

Oxidative stress in cell lines of the MCF10A progression series was determined with Cellular ROS/Superoxide Detection Assay Kit (ab139476, Abcam), which measures total reactive oxygen species and superoxide separately, according to the manufacturer’s protocol. The total ROS level was measured on plate reader with fluorescein filter set. For determination of superoxide signal, micrographs were taken on live cells by fluorescent microscope with Texas Red filter, and the signal intensity per cell was quantified by ImageJ.

### BH_4_ measurement

Cell lines of the MCF10A progression series were cultured until they became confluent in T-175 flasks. After trypsinization, cells were washed 3 times in cold PBS and pelleted. Intercellular BH_4_ levels were measured by the ELISA kit (abx519576, ABBEXA, UK) according to the manufacture’s protocol. Protein concentration and cell number were normalized in the provided sample buffer across the cell lines. The signal intensity was measured in triplicates for each sample at 450 nm.

### NOS knockdown by shRNA

To knock-down each NOS isoform, lentiviral NOS shRNA vectors that target respective sequences were obtained from Origene (Supplementary Table 1). As a control, lentiviral vector that expresses scramble sequence (CAT No: TR30021) was used. Lentiviral particles were produced as described below and applied to MCF10A cells to make stable cell lines.

### Lentivirus production and transduction

Lentivirus production and transduction of target cells were conducted following the guideline by Origene. Briefly, lentivirus vector and packaging plasmid mix (Origene) were transfected into 293FT cells (Invitrogen) using Lipofectamine® 3000. After 48 hrs, medium was harvested, filtered and used to infect target cells with the addition of polybrene (10 μg/ml). After 24 hrs, medium was replaced. At 72 hrs post-infection, puromycin (0.5 μg/ml) was added for selection and maintained throughout the culturing period.

### NOS knockdown by siRNA

To simultaneously knockdown two NOS isoforms, MCF10A cells expressing NOS1-3 shRNA were transfected with NOS1-3 siRNA (Dharmacon, Supplementary Table 2) using Lipofectamine® RNAiMAX Transfection Reagent (ThermoFisher) according to the manufacturer’s guideline.

### Immunohistochemistry

To determine the expression of specific markers, paraffin-embedded sections of mouse mammary tissues were analyzed by immunohistochemistry. Briefly, sections were deparaffinized, hydrated, and treated with antigen unmasking solutions (Vector Laboratories, Inc.) or with Tris-EDTA Buffer (10 mM Tris Base, 1 mM EDTA Solution, 0.05% Tween 20, pH 9.0) heated to 95–100 °C in a pressure cooker. After being blocked with nonimmune goat serum, sections were processed for immunofluorescence staining as described below.

### Immunofluorescence staining and imaging

Immunofluorescence staining/imaging was performed as described previously^24^. Samples were incubated with primary antibody for overnight at 4 degree in a humidified chamber. After intensive washing (three times, 15 min each) in 0.1% BSA, 0.2% Triton-X 100, 0.05% Tween 20, 0.05% NaN3 in PBS, fluorescence-conjugated secondary antibodies (Molecular Probes) were added for 1 hr at room temperature. Nuclei were stained with 0.5 ng/ml DAPI. After mounted with anti-fade solution, epi-fluorescence imaging was performed on Olympus IX70 microscope using CellSens software. Confocal fluorescence imaging with Second Harmonics Generation (SHG) module^96^ was performed on Leica Microsystems TCS SP5 multi-photon laser scanning confocal microscope using Suite Advanced Fluorescence (LAS AF) software.

### Image Analysis

Quantification of fluorescence signal in micrographs was performed with ImageJ software (NIH) referring to the owner’s manual (http://imagej.net/docs/guide/146.html). Briefly, a region of interest (ROI) was determined in reference to an image of DAPI-stained nuclei. For quantification of signal in individual cultured cells, the whole cell was selected as ROI. For quantification of signal in individual organoids in cultures or tissues, each organoid was selected as ROI. For quantification of second harmonics generation (SHG) signal in mammary tissues, the ROI was defined as the periductal ECM/stromal area between the epithelial and adipose layers. For quantification of S-NOC signal in mammary tissues, epithelial layers were selected by setting a threshold range, and the intensity was measured for each gland. For each ROI, the average intensity per pixel was measured, and background intensity was subtracted. For each sample group, at least 50 to 200 measurements were performed. Furthermore, measurement of each sample set was repeated by at least three people, and the results were combined for the final data. The mean value was represented as arbitrary units (AU). The statistical significance of the data was further evaluated using Graphpad Prism Version 5 software (see statistics section).

### Animal Studies

All animal experiments conformed to The Guide for the Care and Use of Laboratory Animals (National Research Council, National Academy Press, Washington, D.C., 2010) and were performed with the approval of the Institutional Animal Care and. Use Committee of the University of Toledo, Toledo, OH (Protocol No: 108658). Three-weeks old female BALB/c (n = 18) mice were obtained from the Jackson Laboratory (Bar Harhor, MN) and housed under a 12 hr light-dark cycle and given regular chow. Starting at the age of 4 weeks old, mice were given intraperitoneal injection of either drug (vehicle: PBS (100 µl), L-arginine (20 mg/kg, 100 µl), or L-NAME (20 mg/kg, 100 µl)) every other day for 6 weeks. Body weight and morbidity of animals were monitored throughout the treatment period. At the end of treatment period, mice were euthanized, and inguinal mammary glands were harvested. Number 4 mammary glands were processed for whole mounting as described^168^, while number 5 glands were processed for paraffin-embedding and sectioning. Whole mount was imaged by *Cytation*™ *5* Cell Imaging Multi-Mode Reader (BioTech Instruments). To determine the gross morphology of glands, paraffin sections were deparaffinized, hydrated and stained with eosin/hematoxyline. Other sections were analyzed by immunohistochemistry and SHG imaging.

### *Ex Vivo* 3D Cultures

Mouse mammary tumors (#4 glands, ~1 cm in diameter, n = 4) were harvested from 18 weeks old female MMTV-PyMT mice (Jackson). Tumors were rinsed in PBS and chopped into ~1 mm × 2 mm × 1 mm fragments, as previously described^169,170^. 1~2 fragments/48 well were plated onto the ECM gel coat (Matrigel) and cultured in DMEM + 10% FBS + 1% penicillin/streptomycin with 4% Matrigel and sepiapterin (0, 20 or 100 μM) for one week with drug replenishment every 2–3 days. Tumors were fixed, paraffin-embedded, sectioned and stained with eosin/hematoxyline.

### Mammosphere assay

MCF10A cell lines were pretreated for 7 days with either vehicle, L-arginine (2.5 mM) or L-NAME (2.5 mM), and then subjected to mammosphere assay as described^120,121,129^. Briefly, the day before experiment, culture plates were coated with poly-HEMA (#3932, Sigma) as previously described^171^. Single cell suspension of 4 × 10^4^ cells were resuspended in 2 ml media from Mammocult Human medium kit (#5620, Stemcell Technologies) with 4 μg/ml heparin and 0.48 μg/ml hydrocortisone, and plated into each well of 6-well plates. Media were replaced every other day, and mammospheres were harvested after 7 days and processed for immunostaining/imaging (spheroids in suspension or paraffin-embedded/sectioned^129^) or FACS analysis (see below). For the mammosphere formation efficiency assay previously described^119–124^, the starting cell density was 10,000 cells per well of 48 well plate (n = 4), and was serially diluted to the level <100 cells per well (10,000, 5000, 2500, 1250, 625, 312, 156, 78; spheres were countable when seeded at the densities ≥1250/48-well for all the treatment groups). After one week of culturing, microscopy images were taken on phase I filter, and spheres larger than 75 μM in diameter were counted using ImageJ. The data are presented as both the number of spheroids formed at each seeding number (Fig. 7C) and mammosphere forming efficiency [(%) = (# of spheroids)/(# of cells seeded) × 100] for each seeding density (Supplementary Fig. 7B)^119–124^. For limiting dilution analysis previously described^126^, the starting cell density was 1,600 cells per well of 96 well plate (n = 8), and was serially diluted to 200 cells per well. Each well was examined under light microscope and scored as either “ + ” (spheres present) or “−” (no spheres present). The fraction of “−” wells (1 − P) was calculated as the number of “−” wells/total wells for each condition. Ln (fraction of “−” wells) was plotted in the Y-axis against the number cells seeded in the X-axis, where the of best fit was drown to intersect at the origin. The sphere forming frequency corresponds to −(slope) of the line (see the equation below^126^).

$${\rm{P}}={\rm{1}}-{{\rm{e}}}^{{\rm{mD}}},{\rm{P}}:{\rm{probably}}\,{\rm{of}}\,{\rm{sphere}}\,{\rm{formation}};{\rm{m}}:{\rm{slope}};{\rm{D}}:{\rm{seeding}}\,{\rm{number}}$$

$$\mathrm{Ln}\,(1-{\rm{P}})=\,\mathrm{Ln}\,(\mathrm{fraction}\,{\rm{of}}\,\mbox{''}-\mathrm{wells})={\rm{mD}}$$

$${\rm{Sphere}}\,{\rm{forming}}\,{\rm{frequency}}=-\,({\rm{slope}})=-{\rm{m}}$$

### Flow cytometric (FACS) analysis of mammospheres

FACS analysis of mammospheres was performed as described with slight modifications^172^. Briefly, mammospheres were collected by centrifugation, resuspended in 200 μL of 1 × 0.25% trypsin-EDTA and incubated for 5 minutes to dissociate cells. Cells were washed and resuspended in the growth medium and rotated at 37 °C in a hybridization oven (15 rpm) for 8 hours to recover cell-surface components^173^. 1 × 10^5^ cells (100 μl in PBS) were incubated with 1 μg of anti-CD44 and anti-CD24 antibodies or isotype control, followed by the respective fluorophore-conjugated secondary antibodies at room temperature. Cells were collected by centrifugation and resuspended in PBS + BSA (1%) before loading onto BD Biosciences FACSARIA IIU operated with BD FACSDIVA software. The fluorescein isothiocyanate (FITC) was excited at 488 nm and emission recorded in the 530/30 filter channel, while Alexa Fluor 594 fluorophore was excited at 488 nm and emission recorded in the 610/20 filter channel. Compensation values for FITC and Alexa Fluor 594 fluorescence overlap were established using compensation controls and copied to all subsequent analyses. A total of 1000 events were recorded for each sample. Isotype controls were used to set threshold gates.

### Statistics

All the experiments were performed in replicates (n > = 3 for *in vitro* experiments; n > 6 for *in vivo* experiments) ensuring the adequate statistical power as done previously^174^. Unless otherwise indicated, statistical significance of the mean difference was tested by two-tailed t-tests (parametric) using Graphpad Prism Version 5 software. P-values of 0.05 or less were considered significant. Average results of multiple experiments (n > = 3) are presented as the arithmetic mean ± SEM.

## Supplementary information

Reduced Basal Nitric Oxide Production Induces Precancerous Mammary Lesions via ERBB2 and TGFβ
