# Supplementary material for: Reduced Basal Nitric Oxide Production Induces Precancerous Mammary Lesions via ERBB2 and TGFβ
Source: Sci Rep. 2019 Apr 30;9:6688. doi: 10.1038/s41598-019-43239-x (PMC6491486; doi:10.1038/s41598-019-43239-x)
Supplement: Supplementary file 1 — Reduced Basal Nitric Oxide Production Induces Precancerous Mammary Lesions via ERBB2 and TGFβ [file 41598_2019_43239_MOESM1_ESM.pdf]

Reduced Basal Nitric Oxide Production Induces Precancerous Mammary Lesions via ERBB2 and TGFβ

Gang Ren[1*], Xunzhen Zheng[1*], Matthew Bommarito[1], Samantha Metzger[1], Yashna Walia[1], Joshua Letson[1], Allen Schroering[2], Andrea Kalinoski[2], David Weaver[2], Christopher Figy[1], Kam Yeung[1], Saori Furuta[1**]

[1]University of Toledo Health Science Campus, College of Medicine and Life Sciences, Department of Cancer Biology, 3000 Arlington Ave. MS1010, Toledo, OH, USA

[2]University of Toledo Health Science Campus, College of Medicine and Life Sciences, Department of Surgery, 3000 Arlington Ave. MS1008, Toledo, OH, USA

[*]These authors contributed equally.

[**]Corresponding author: saori.furuta@utoledo.edu

Supplementary Figures and legeds

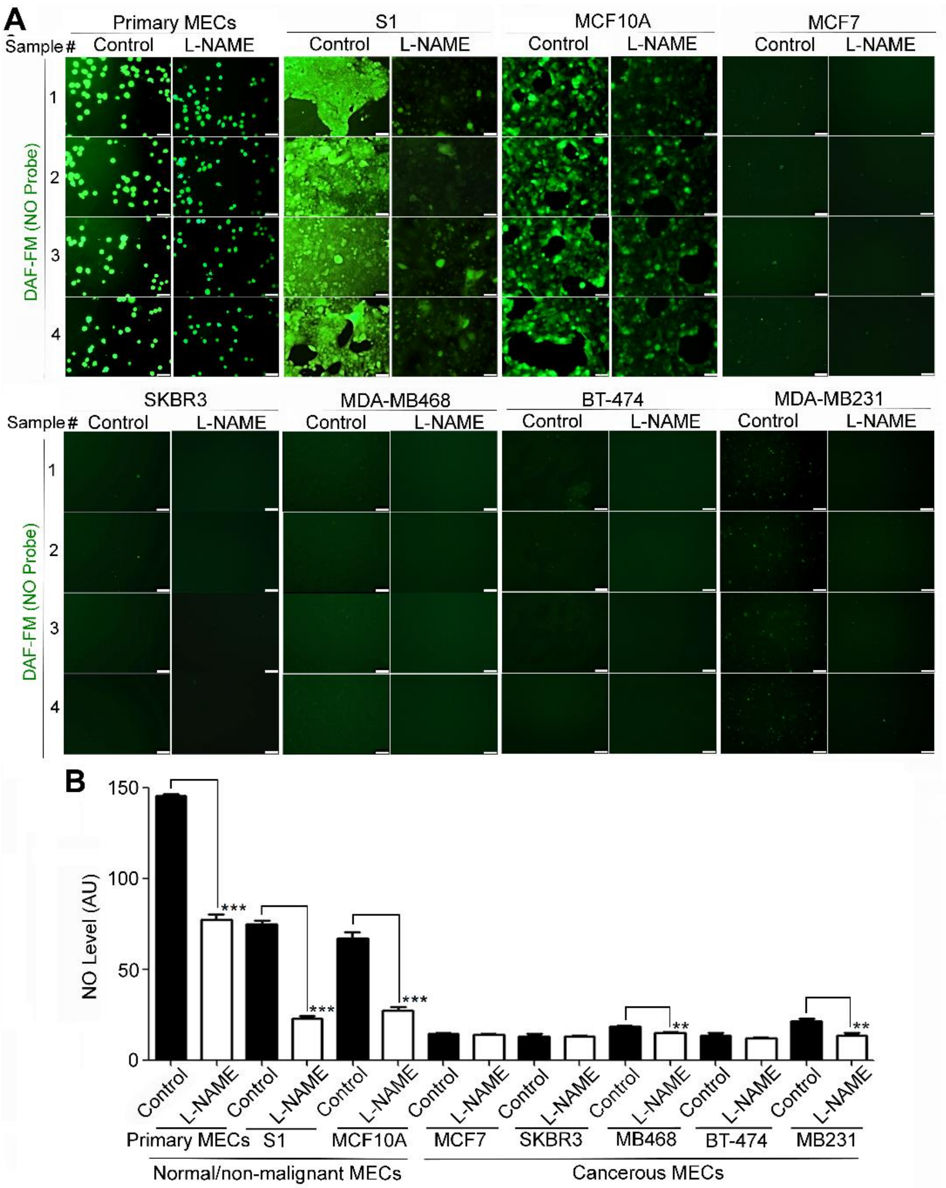

Supplementary Figure 1: Production of the basal level of nitric oxide (NO) is prominent in normal/non-malignant MECs, but abrogated in cancerous MECs in 3D cultures. A) Micrographs of a panel of normal/non-malignant (primary MECs, HMT3522-S1 and MCF10A) and cancerous breast cell lines (MCF7, SKBR3, MDA-MB468, BT474 and MDA-MB231) cultured with 5% Matrigel drip and stained with DAF-FM DA. To test the specificity of the signal, NOS inhibitor, L-NAME (2.5 mM) was applied. Scale bars: 20 µm. **B**) Quantification of DAF-FM signals/cell. Error bars: mean ± STDEV. ** $p<0.01$ and *** $p<0.001$.

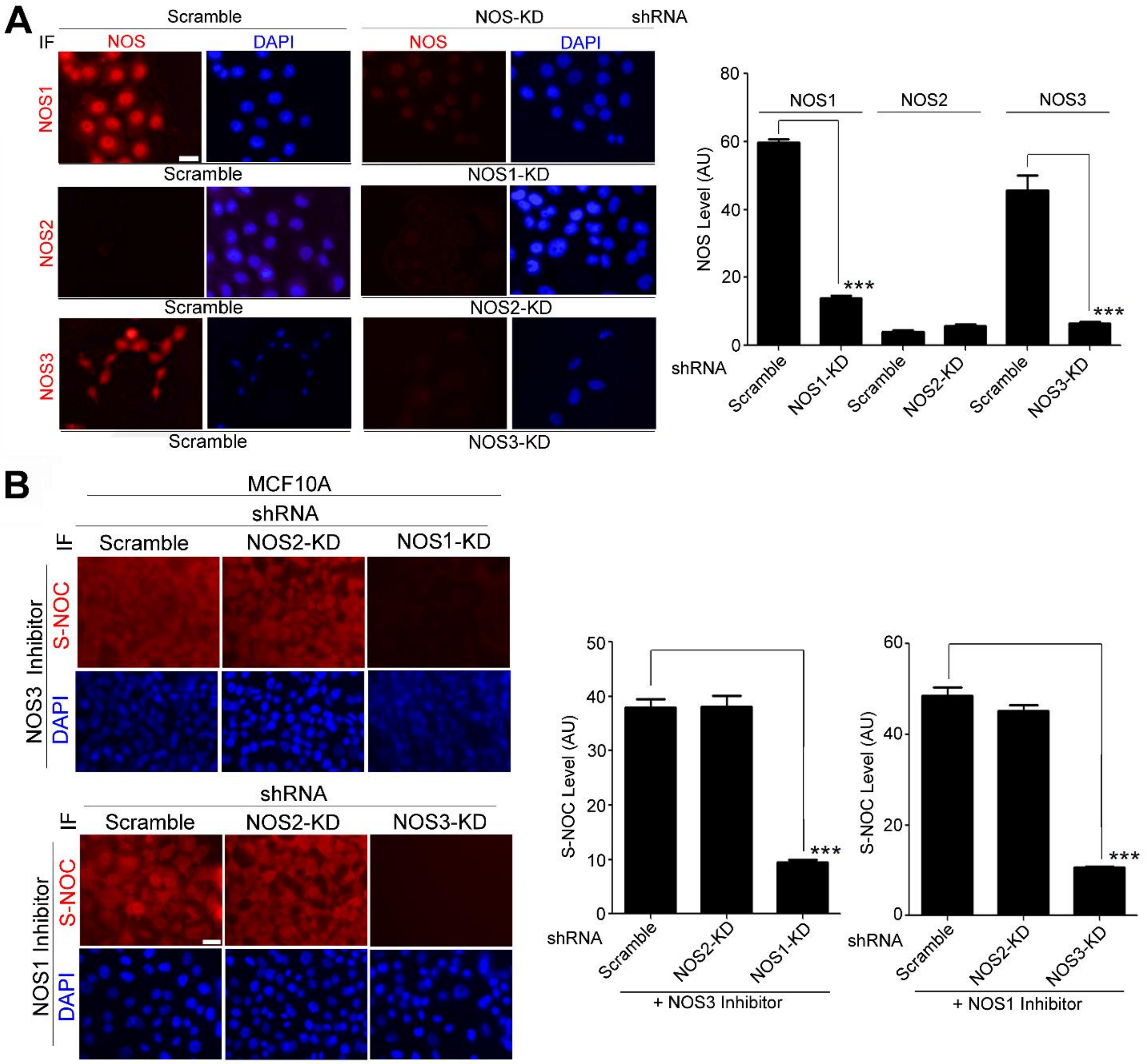

Supplementary Figure 2: NOS-1 and NOS-3 are involved in the production of basal NO in mammary epithelial cells. A) Validation of shRNA knockdown of NOS1-3 in MCF10A cells. (Left) MCF10A cells immunostained for NOS-1, -2 or -3 after transduced with scramble or NOS-1, -2 or -3 shRNA lentivirus, respectively. (Right) Quantification of NOS-1, -2 and -3 levels/cell after shRNA treatment. **B**) S-NOC level in MCF10A cells expressing scramble, NOS-2 or -1 shRNA and treated with NOS-3 inhibitor (top 2 rows) or cells expressing scramble, NOS-2 or -3 shRNA and treated with NOS-1 inhibitor (bottom 2 rows). (Right) Quantification of S-NOC level in MCF10A cells after shRNA + inhibitor treatment.

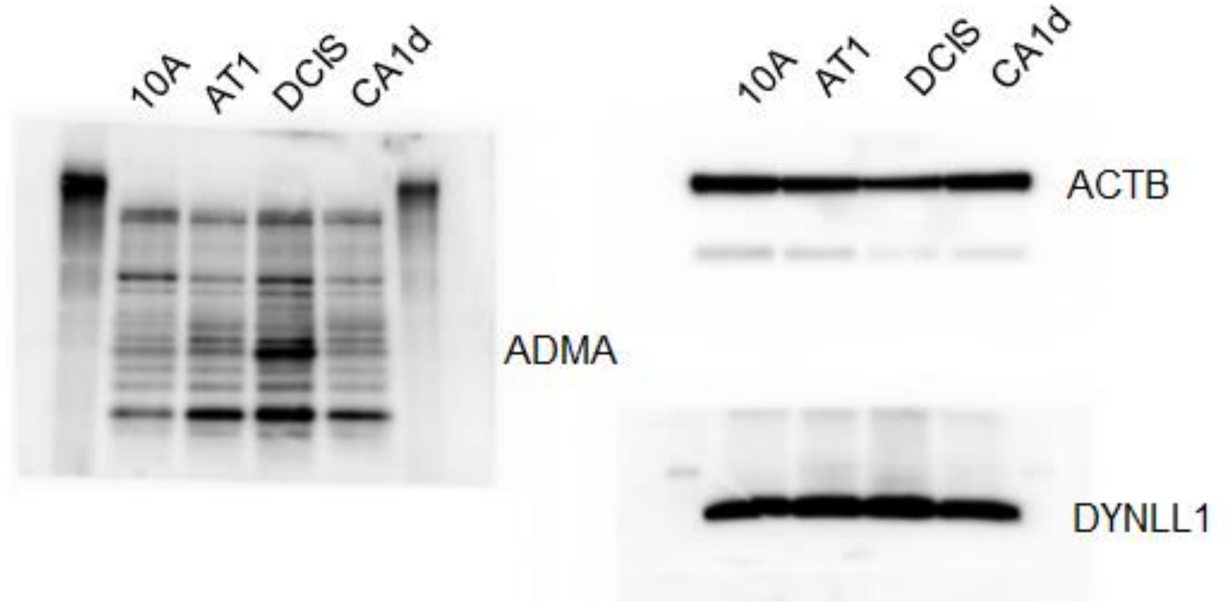

Supplementary Figure 3. Raw gel images of western blot in **Figure 3E** showing levels of Endogenous NOS inhibitors ADMA (left)and DYNLL1 (right) in MCF10A progression series. β-actin (ACTB) serves as a loading control.

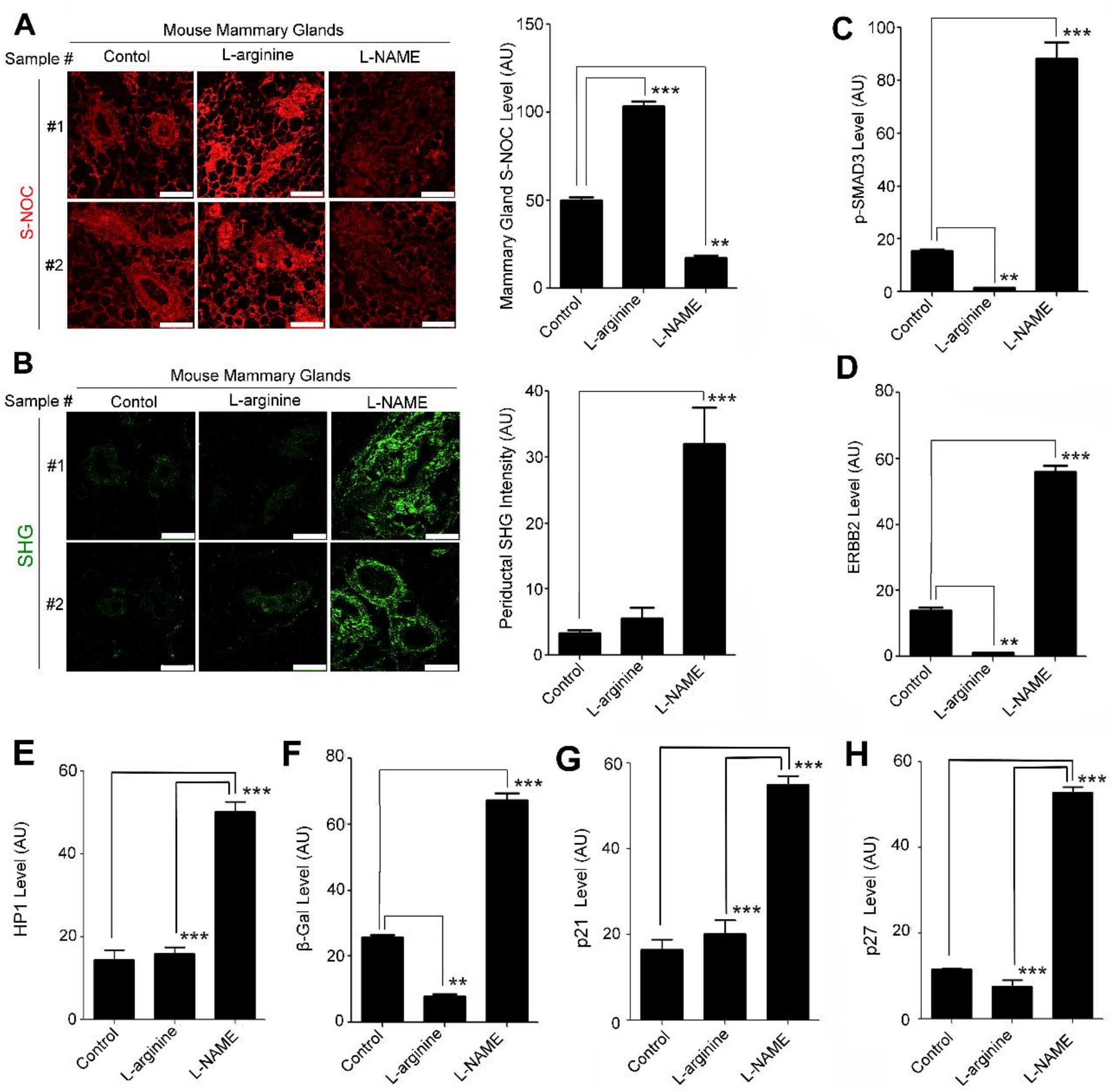

Supplementary Figure 4: Inhibition of NO production in developing mouse mammary glands stiffens the ECM, upregulates TGFβ and ERBB2 and induces senescence. A) (Left) S-NOC staining of mammary glands treated with control (PBS), L-arginine or L-NAME for 6 weeks. Scale bars: 100 μm. (Right) Quantification of S-NOC signal/cell in mammary epithelia. **B**) (Left) Second harmonics generation (SHG) imaging to visualize collagen in drug-treated mammary glands. Scale bars: 100 μm. (Right) Quantification of SHG signal in the periductal regions. **C**) The level of phospho-SMAD3 in mouse mammary glands as in **Figure 4B**. **D-F**) The level of ERBB2 (**D**), HP1 (**E**), β-Gal (**F**), p21 (**G**) or p27 (**H**) in mouse mammary glands as in **Figure 4D**. Error bars: mean ± STDEV. *** $p<0.001$.

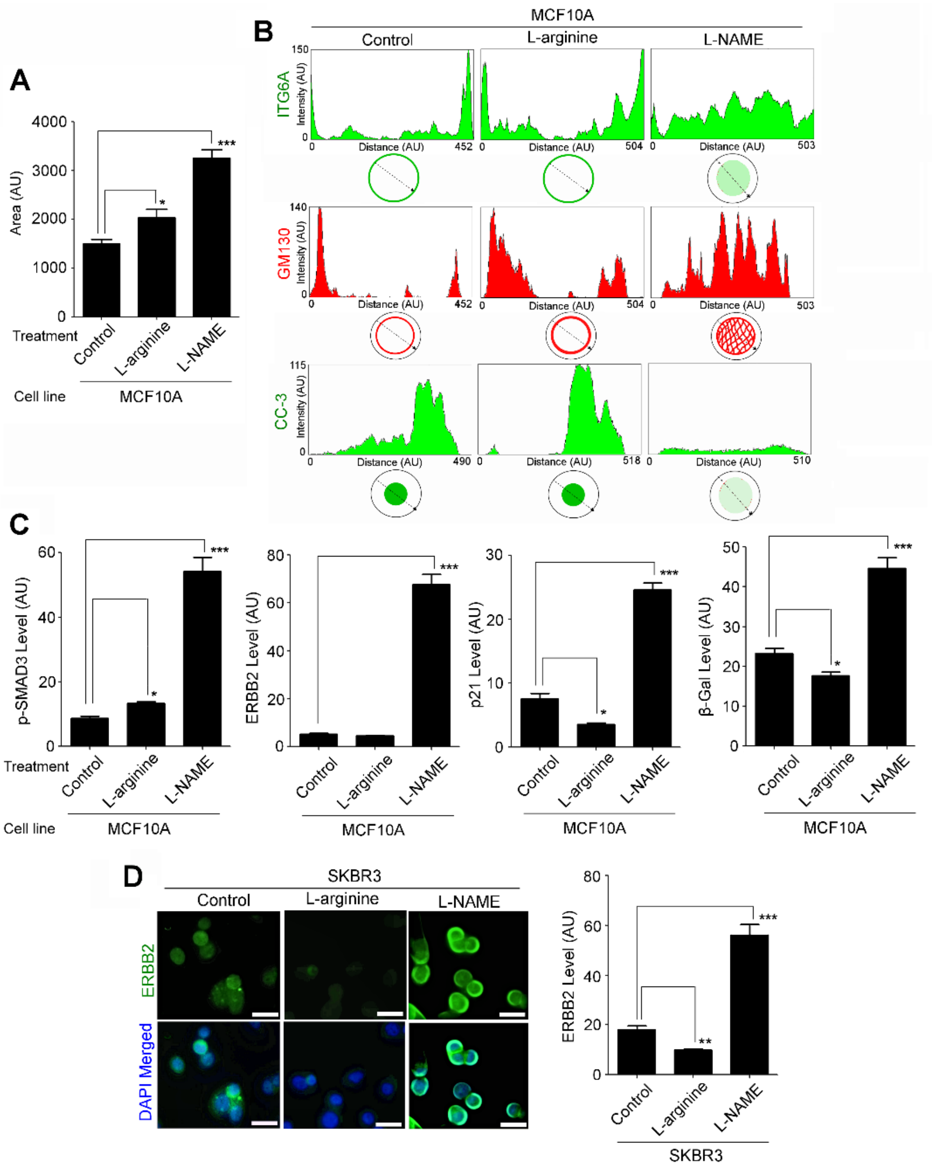

Supplementary Figure 5: Deprivation of NO in non-malignant mammary epithelial cells in 3D cultures increases colony size and upregulates TGFβ, ERBB2 and senescence markers. **A**) Colony size of MCF10A cells cultivated in 3D ECM under the treatment of control (PBS), L-arginine or L-NAME for 3 weeks as in **Figure 5A**. **B**) Plot profiles (ImageJ) of polarity markers, ITGA6 (basal), GM130 (apical) and CC-3 (a marker for the lumen), across individual drug-treated MCF10A cells in 3D cultures as in **Figure 5A**. **C**) The levels of phospho-SMAD3, ERBB2, p21 and β-Gal in drug-treated MCF10A cells cultivated in 3D ECM as in **Figure 5B**. **D**) Left: Representative images of ERBB2 staining of SKBR3 breast cancer cells treated with control, L-arginine or L-NAME overnight. Nuclei were counterstained with DAPI (blue). Scale bars: 20 µm. Right: ERBB2 level in drug-treated SKBR3 cells. Error bars: mean ± STDEV. * $p<0.05$; ** $p<0.01$; and *** $p<0.001$.

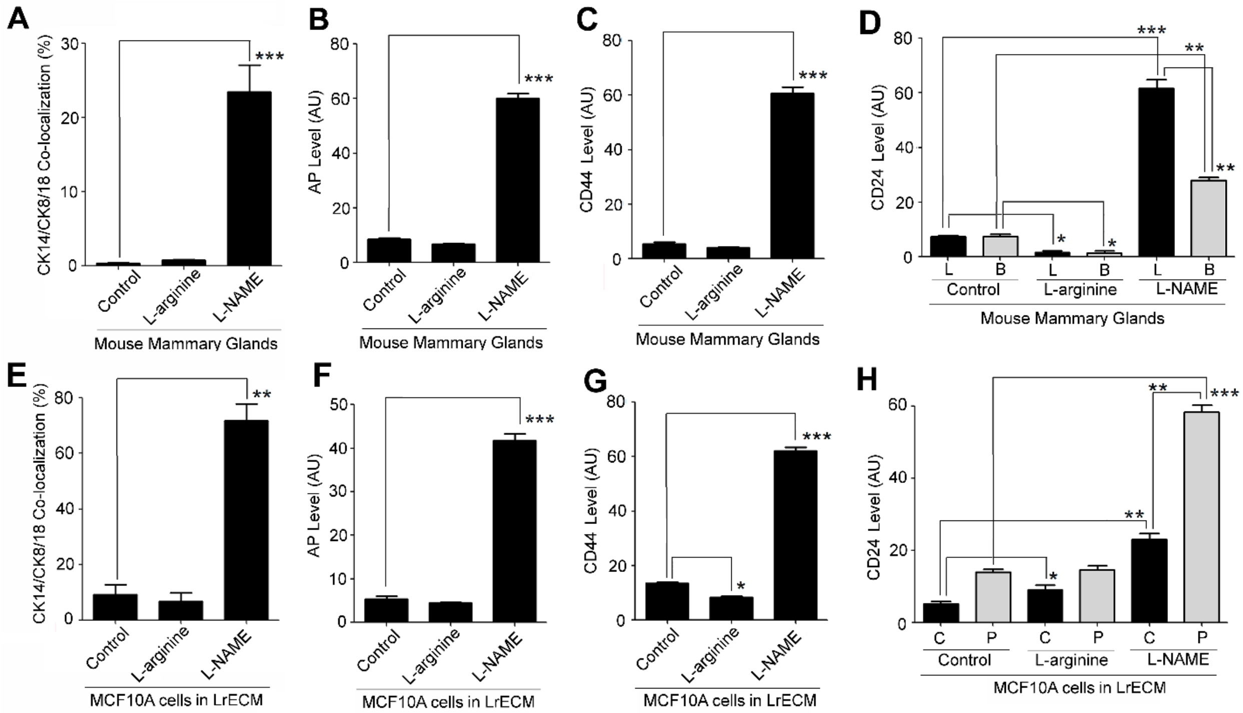

Supplementary Figure 6: Deprivation of NO induces expression of stem cell markers in mammary epithelial cells both *in vivo* and in 3D ECM cultures. A) Percentage of the epithelia in the mammary gland showing CK14 and CK8/18 co-localization after treatment with control (PBS), L-arginine or L-NAME as in **Figure 6A**. **B-D**) The levels of stem cell markers, alkaline phosphatase (AP) (**B**), CD44 (**C**) and CD24 (**D**) in drug-treated mammary glands as in **Figure 6B**. CD44 expression was measured if membranous; CD24 level was measured for luminal (L) vs. basal (B) expression[1]. **E**) Percentage of 3D colonies of MCF10A cells showing CK14 and CK8/18 co-localization after drug treatment as in **Figure 6C**. **F-H**) The level of AP (**F**), CD44 (**G**) or CD24 (**H**) in drug-treated MCF10A cells cultivated in 3D ECM as in **Figure 6D**. CD44 expression was measured if membranous; CD24 level was measured for central (C) vs. peripheral (P) expression. Error bars: mean ± STDEV. * $p<0.05$, ** $p<0.01$ and *** $p<0.001$.

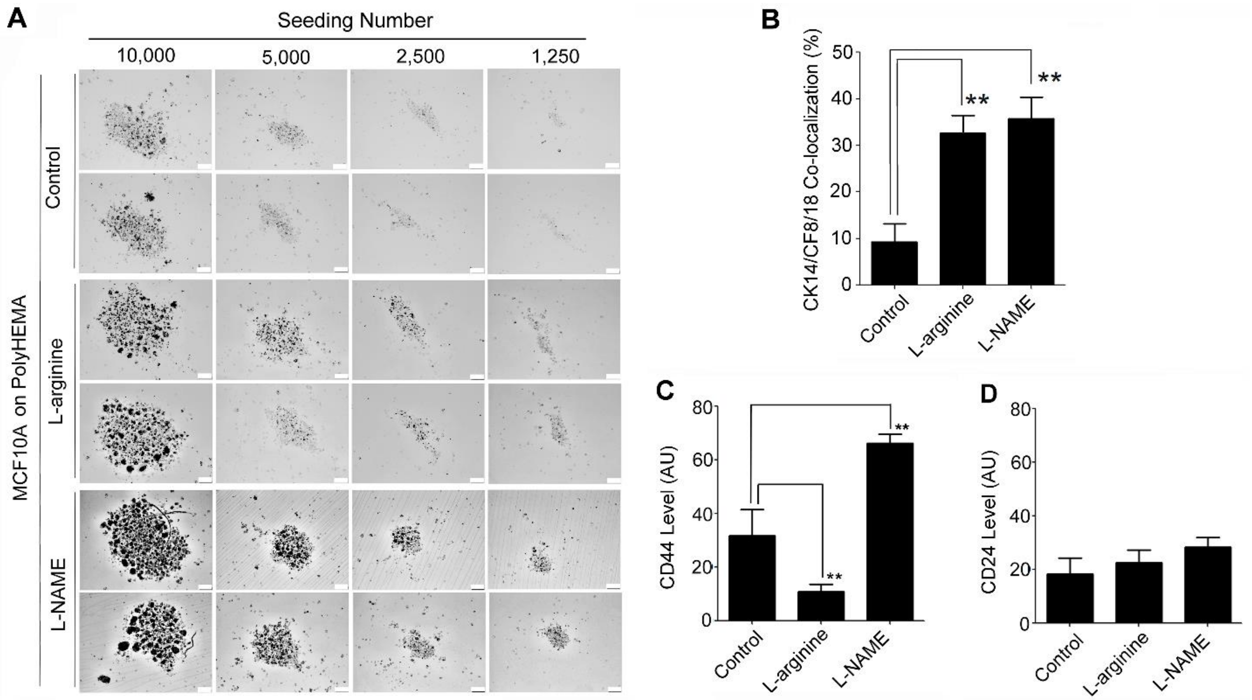

Supplementary Figure 7: Deprivation of NO elevates mammosphere forming potential of non-malignant mammary epithelial cells. A) Representative images of mammospheres derived from MCF10A cells seeded at different cell numbers and grown on PolyHEMA under the treatment of control (PBS), L-arginine or L-NAME. Scale bars: 200 µm. **B**) Percentage of cells in each mammosphere showing CK14 and CK8/18 co-localization after treatment with control (PBS), L-arginine or L-NAME as in **Figure 7E**. **C-D**) The levels of mammary stem cell markers, CD44 (**C**) and CD24 (**D**), in drug-treated mammospheres determined by immunofluorescence imaging as in **Fig. 7G**. Error bars: mean ± STDEV. *** p<0.001.

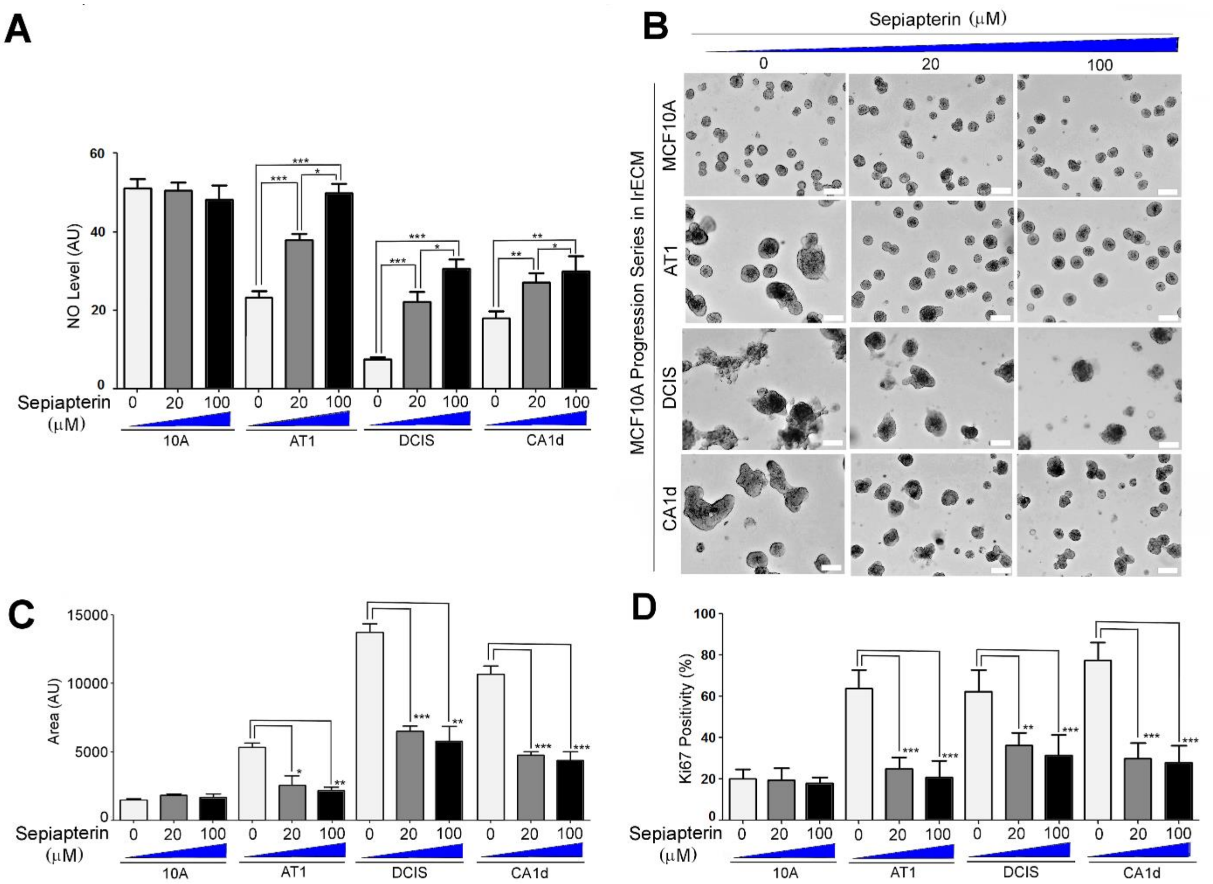

Supplementary Figure 8: Suppressive effects of sepiapterin on precancerous and cancerous cells are concentration-independent. **A-D**) Cell lines of the MCF10A progression series cultured in 5% Matrigel drip with a different concentration of sepiapterin (0, 20 or 100 μM). **A**) NO level determined with the NO probe DAF-FM DA. **B**) Representative images of 3D colonies. **C**) Colony size. **D**) Percent of Ki67-positive cells/colony. Error bars: mean ± STDEV. ** p<0.01; *** p<0.001.

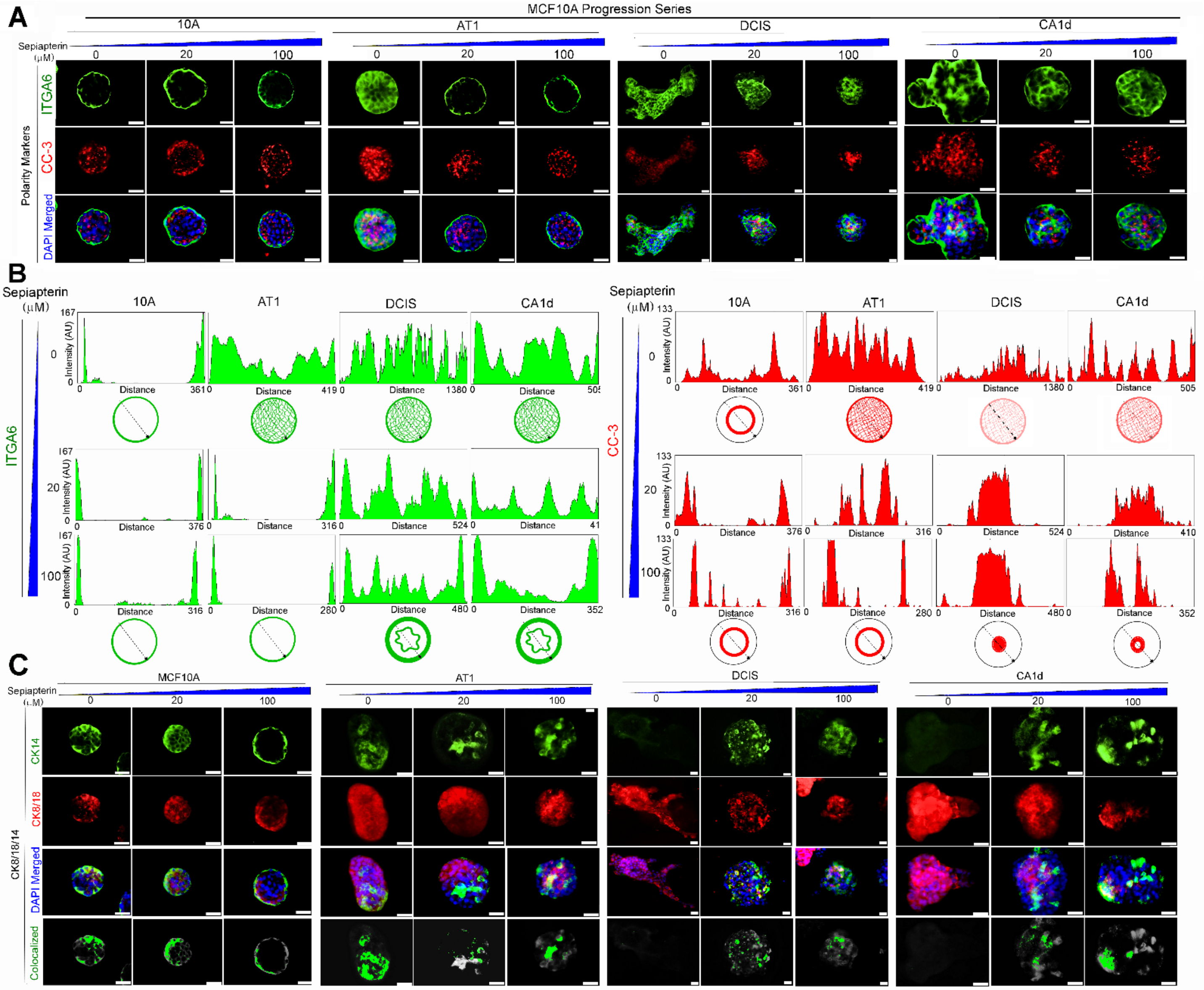

Supplementry Figure 9: Sepiapterin normalizes the polarity of precancerous and cancerous cell lines of the MCF10A progression series in 3D ECM cultures. **A**) Representative images of the MCF10A progression series cultivated in 3D ECM with a different concentration of sepiapterin (0, 20 or 100 μM) and stained for polarity markers, ITGA6 (basal) and CC-3 (lumen). Nuclei were counterstained with DAPI (blue). Scale bars: 20 μm. **B**) Plot profiles of ITGA6 and CC-3 across individual 3D colonies of the progression series cultivated as in **A**). Note that sepiapterin has no effect on non-malignant cells, while it, at least partially, restores the polarity of precancerous and cancerous cells at the concentrations of both 20 and 100 μM. **C**) Cell lines of MCF10A progression series were cultivated as in **A**) and stained for lineage markers, CK14 (basal cell) and CK8/18 (luminal cell). Co-localization of CK14 and CK8/18 was captured with ImageJ. Note that sepiapterin restores the basal cell marker CK14 in DCIS and CA1d cells at the concentrations of both 20 and 100 μM.

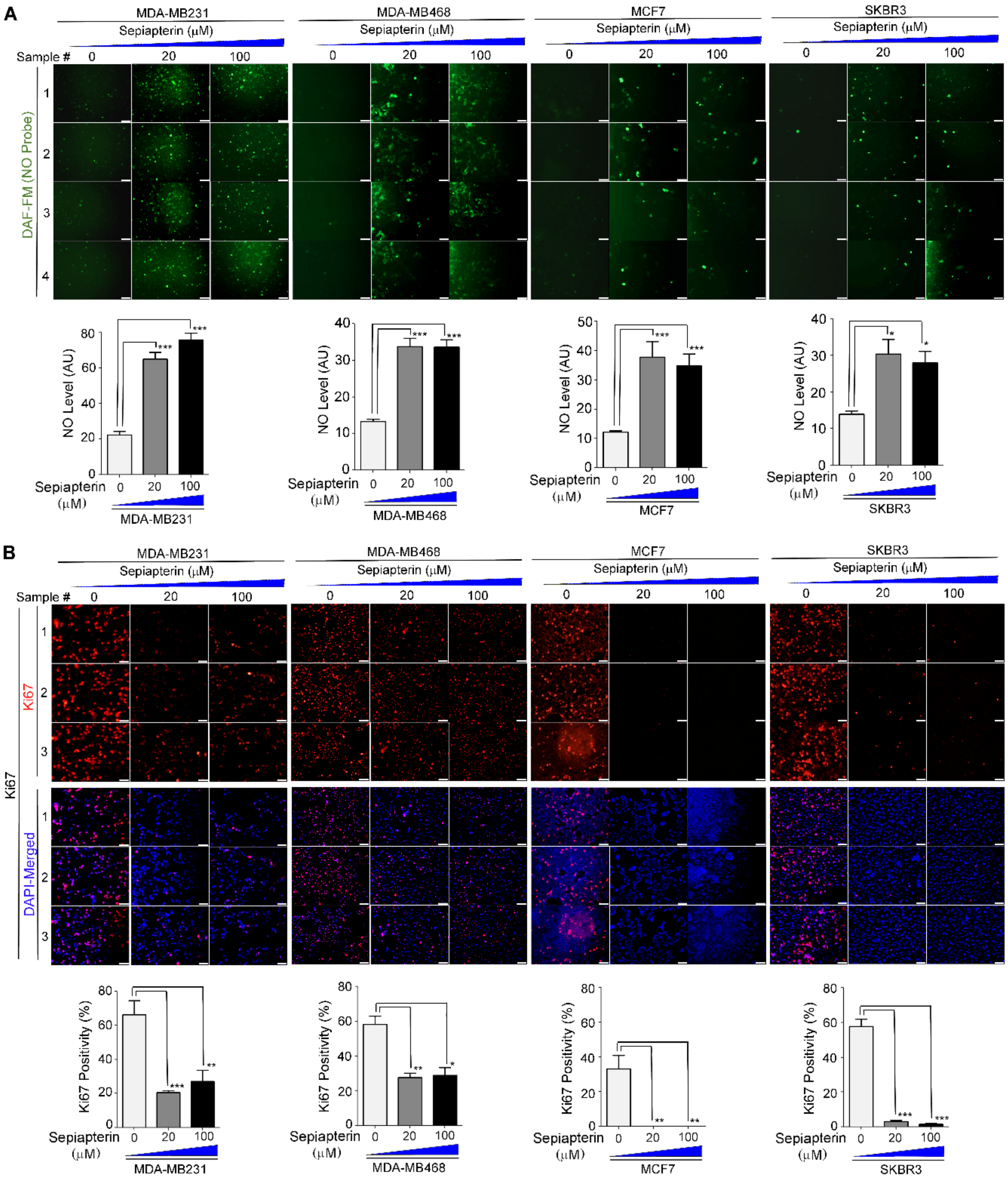

Supplementary Figure 10: Sepiapterin elevates the basal NO level and suppresses proliferation of breast cancer cells cultured in lrECM in a threshold-dependent manner. **A**) (Top) Micrographs of different breast cancer cells (basal: MDA-MB231 and MDA-MB468; luminal: MCF7 and SKBR3) cultured in 5% Matrigel drip with a different concentration of sepiapterin (0, 20 or 100 μM) for 2 hours and stained with the NO probe DAF-FM DA. (Bottom) Quantification of DAF-FM signal/cell. **B**) (Top, top 3 rows) Different breast cancer cells cultured as in **A**) and stained for Ki67. (Top, bottom 3 rows) DAPI-merged micrographs. (Bottom) Percentage Ki67-positive cells. Error bars: mean ± STDEV. * p<0.05, ** p<0.01 and *** p<0.001.

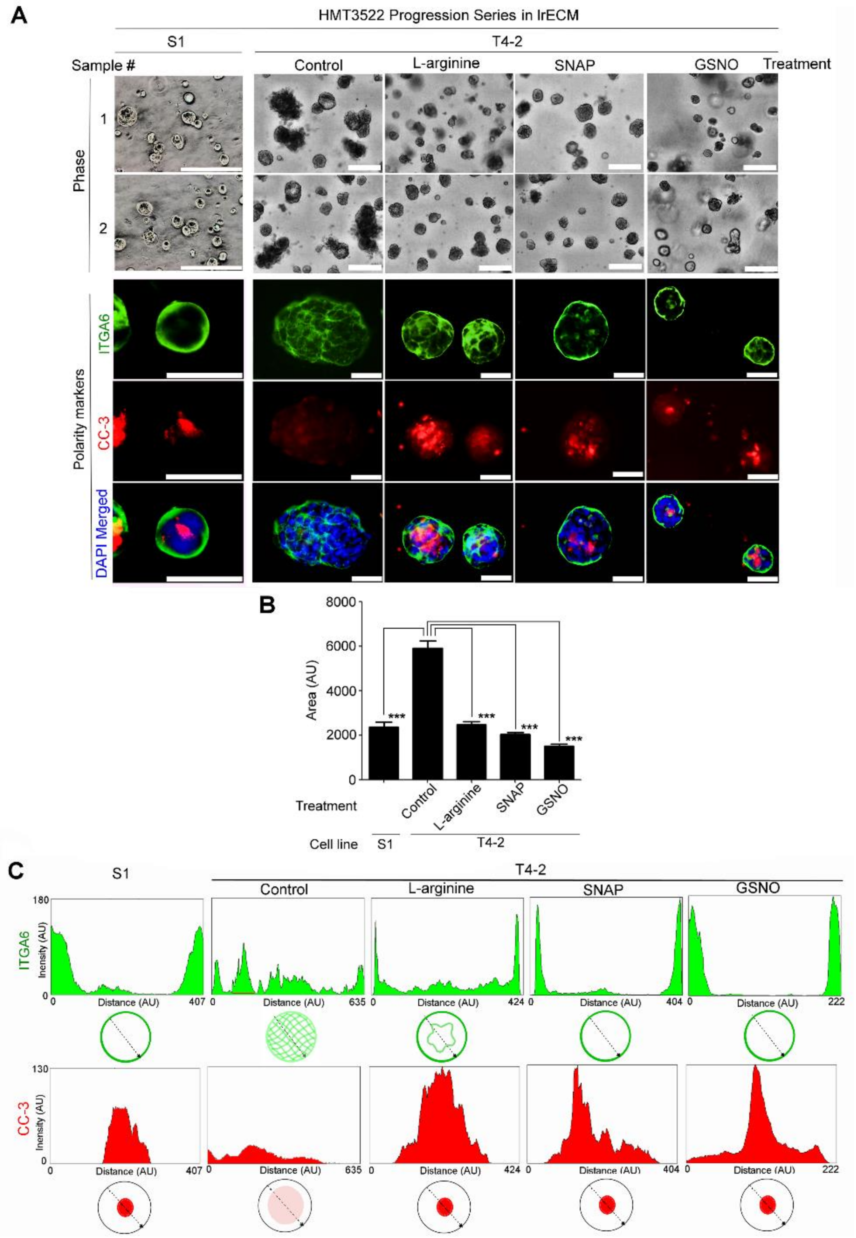

Supplementary Figure 11: NO donors, SNAP and GSNO, at a low concentration ameliorate malignant phenotype of another breast cancer progression series, HMT-3522. A) Top 2 rows: Representative phase images of the HMT3522 progression series (non-malignant S1 and malignant T4-2)[2] in 3D ECM cultures, where T4-2 cells were treated with control (PBS), L-arginine, SNAP (2.5 µM) or GSNO (2.5 µM). Bottom 3 rows: S1 and drug-treated T4-2 cells stained for polarity markers, ITGA6 (basal) and CC-3 (a marker for the lumen). Nuclei were counterstained with DAPI (blue). Scale bars: 200 µm (top 2 row); 50 µm (bottom 3 rows). **B**) 3D colony sizes of S1 and drug-treated T4-2 cells. **C**) Plot profiles of ITGA6 and CC-3 signals across individual 3D colonies of S1 and drug-treated T4-2 cells as in **A)**. Note the restoration of the S1 cell-like polarity and growth-arrested phenotype in T4-2 cells by treatment with 2.5 µM SNAP or GSNO. Error bars: mean ± STDEV. *** p<0.001. (See the effects of different concentrations of SNAP and GSNO in **Supplementary Figures 12A-C**.)

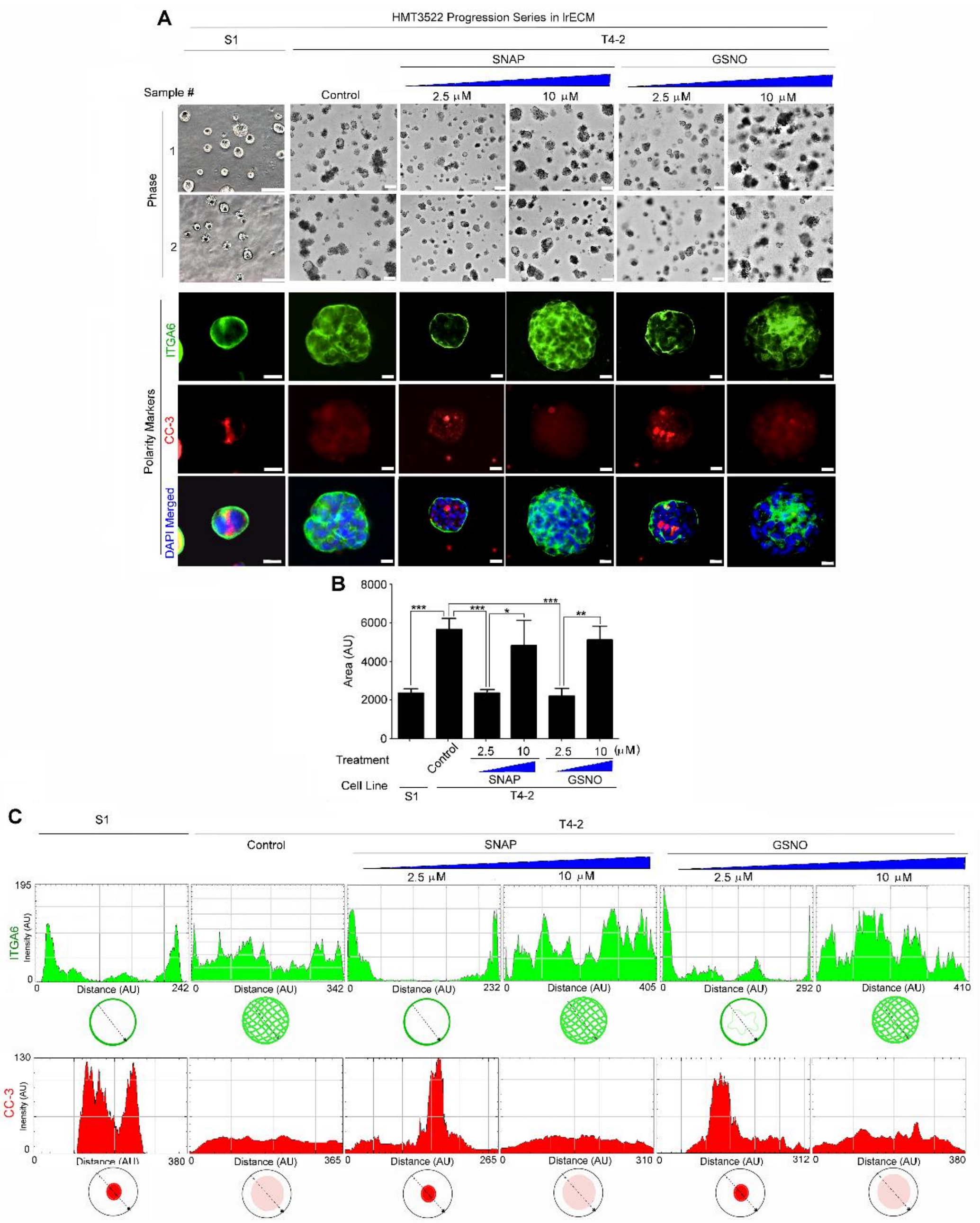

Supplementary Figure 12: Suppressive effects of NO donors, SNAP and GSNO, on breast cancer cells are concentration-dependent. **A**) Top 2 rows: Representative phase images of the HMT-3522 progression series (non-malignant S1 and malignant T4-2) in 3D cultures, where T4-2 cells were treated with control (PBS), SNAP (2.5 or 10 μM) or GSNO (2.5 or 10 μM). Bottom 3 rows: S1 and drug-treated T4-2 cells stained for polarity markers, ITGA6 (basal) and CC-3 (a marker for the lumen). Nuclei were counterstained with DAPI (blue). Scale bars: 200 μm (top 2 row); 50 μm (bottom 3 rows). **B**) 3D colony sizes of S1 and drug-treated T4-2 cells. **C**) Plot profiles of ITGA6 and CC-3 signals across individual 3D colonies of S1 and drug-treated T4-2 cells as in **A)**. Note that while the lower level (2.5 μM) of SNAP and GSNO restores the S1 cell-like polarity and growth-arrested phenotype in T4-2 cells, the higher level (10 μM) of the NO donors shows no such effects. Error bars: mean ± STDEV. *** $p<0.001$.

Supplementary Tables

NOS1 shRNAs (CAT No:TL311142)		
Sequences	Tube ID	Species Specificity (human, mouse, rat)
**GCTACAAGGTCCGATTCAACAGCGTCTCC**	**TL311142A / TL311142A**	**H,M**
GCCTTCAAGTACTACCTGGACATCACCAC	TL311142B / TL311142B	H,M
GTGGTCACCAGCACCTTTGGCAATGGAGA	TL311142C / TL311142C	H,M
TTCCACCAGGAGATGCTCAACTACCGGCT	TL311142D / TL311142D	H,M
NOS2 shRNAs (CAT No:TL302918)		
Sequences	Tube ID	Species Specificity (human, mouse, rat)
CTCTGGAAAGACCAGGCTGTCGTTGAGAT	TL302918A / TL302918A	H
CGGCAGAATCTACAAAGTCCGACATCCAG	TL302918B / TL302918B	H
GCCAAGAACGTGTTCACCATGAGGCTCAA	TL302918C / TL302918C	H
**AGGTTGTCTGCATGGATAAGTACAGGCTG**	**TL302918D / TL302918D**	**H**
NOS3 shRNA (CAT No: TL320432)		
Sequences	Tube ID	Species Specificity (human, mouse, rat)
CGAGGAGACTTCCGAATCTGGAACAGCCA	TL320432A / TL320432A	H
CGAGTGAAGGCGACAATCCTGTATGGCTC	TL320432B / TL320432B	H
ATCCGCTTCAACAGCATCTCCTGCTCAGA	TL320432C / TL320432C	H
**AAGATGTTCCAGGCTACAATCCGCTCAGT**	**TL320432D / TL320432D**	**H**

Supplementary Table 1: Sequences of NOS shRNAs used in this study.
For each NOS isoform, 4 different shRNA sequences were compared for the knock-down efficiency, and one shRNA sequence was chosen for further study (indicated in **bold**).

ON-TARGETplus Human NOS1 (4842) siRNA - SMARTpool, 5 nmol
GAACACGCAUGUCUGGAAA
CAACAGCGGCAAUUUGAUA
UGAAGGAGCGGGUCAGUAA
GAAAUUCGGCUGUGCUUUG
ON-TARGETplus Human NOS2 (4843) siRNA - SMARTpool, 5 nmol
CGCCUUUGCUCAUGACAUU
UUGCUGUGCUCCAUAGUUU
GCUGCAGAAUCCUUCAUGA
UCGAAUUUGUCAACCAAUA
ON-TARGETplus Human NOS3 (4846) siRNA - SMARTpool, 5 nmol
CAGCACAAGAGUUAUAAGA
CACAGGAAAUGUUCACCUA
GCUCGGCCAUCACAGUGUU
GCUCCCAACUUGACCAUCU
ON-TARGETplus Non-targeting siRNA #1, 20 nmol
UGGUUUACAUGUCGACUAA

Supplementary Table 2: Sequences of NOS siRNAs used in this study.
